# GWAS Combined with WGCNA of Transcriptome and Metabolome to Excavate Key Candidate Genes for Rice Anaerobic Germination

**DOI:** 10.1186/s12284-023-00667-8

**Published:** 2023-10-31

**Authors:** Dandan Li, Kai Liu, Chuanchao Zhao, Siyi Liang, Jing Yang, Ziai Peng, Aoyun Xia, Meng Yang, Lixin Luo, Cuihong Huang, Jiafeng Wang, Ming Huang, Wuming Xiao, Hui Wang, Ling Su, Tao Guo

**Affiliations:** 1 National Engineering Research Center of Plant Space Breeding, South China Agricultural University, Guangzhou, 510642 China; 2 Jiangxi Academy of Eco-environmental Sciences and Planning, Nanchang, 330039 China

**Keywords:** Rice, Genome-wide association analysis (GWAS), Metabolomics, Transcriptomics, WGCNA, Anaerobic germination

## Abstract

Direct seeding of rice is a lightweight and simple cultivation method, which can effectively promote rice production. Anaerobic germination tolerance is one of the main traits of rice adaptability to direct seeding. The mining of related genetic loci, analysis of anaerobic traits and screening of tolerance genes provided valuable genetic resources for improving the anaerobic germination ability of direct seeding rice. This study conducted a dynamic genome-wide association study (GWAS) based on coleoptile-related traits of 591 rice natural populations, and a total of 317 SNP sites were detected. Integrated dynamic widely targeted metabolomics analysis, we found that xanthine, l-alanine and GABA may be key biomarkers that are sensitive and respond strongly to hypoxic stress perception. By WGCNA analysis of targeted metabolomics and transcriptomics, a total of 3 modules were obtained that were significantly correlated with the above three marker metabolites, namely dark green, dark gray and light green modules, respectively, and several key structural genes of *OsAlaAT1*, *OsGAD4*, *OsAAH* and *Os09g0424600* that may affect hypoxic germination were screened from the 3 modules. Among them, *OsAlaAT1* (*Os10g0390500*), located in Chr10-12877840, which is within the GWAS location range of CVAN3d, is considered to be a more reliable candidate gene. Overall, in addition to providing new insight into the metabolic regulation of l-alanine, GABA and xanthine during hypoxic germination of rice. This study also provided a reference for the basic theoretical research and breeding application research on the related traits of anaerobic germination in direct-seeding rice.

**Supplementary Information:**

The online version contains supplementary material available at 10.1186/s12284-023-00667-8.

## Introduction

Rice (*Oryza sativa* L.) is the staple food for more than half of the world's population. Producing enough food to meet the needs of the rapidly growing population is one of the most important challenges in the twenty-first century (Yu et al. 2020). Seed germination and seedling establishment, as key events in plant life activities, are important factors influencing the yield of direct seeding in rice (Gommers and Monte 2017). Seed germination is a key stage in the life cycle of cereal crops, starting from the absorption of water by dry seeds to the end of radicle and embryo protrusion (Yang et al. 2010). Oxygen (O_2_) is necessary for aerobic oxidative respiration, which generates energy in all organisms (Yu et al. 2020). Hypoxic stress caused by severe or unexpected flood events results in insufficient energy and slow and uneven seed germination, which eventually lead to serious crop loss (Voesenek and Bailey-Serres 2009).

Unlike seeds of other cereal crops, rice seeds can germinate successfully under low oxygen (limited O_2_) conditions or even in the absence of O_2_ (Lee et al. 2009). It was found that “escape and metabolic adaptation” were the main strategies for rapid anaerobic germination (AG) of rice under submerged conditions. That is, the hollow coleoptile rapidly elongates to the aerated water surface, allowing O_2_ to diffuse into the endosperm to hydrolyze nutrients and support the vigorous establishment of seedlings. The transcriptional profile of rice coleoptiles germinated under hypoxia showed that the genes encoding enzymes for sucrose and starch metabolism, glycolysis, fermentation and cell expansion, heat shock protein and ethylene response factor were significantly upregulated, while the genes encoding enzymes requiring O_2_ for activity were significantly downregulated (Lasanthi-Kudahettige et al. 2007). These transcriptomic data related to various metabolic and signaling pathways indicate the physiological and growth reprogramming of rice seed germination under hypoxic stress.

In addition to the anaerobic reactions triggered by sucrose, starch metabolism and glycolysis, the production and accumulation of some amino acids are also common reactions in plants under hypoxic stress (Miyashita et al. 2007). De novo synthesis of approximately 20 anaerobic polypeptides (ANPs) (Sachs et al. 1980; Galili et al. 2014) triggered a metabolic transition from oxidative phosphorylation to anaerobic fermentation. These ANPs, including enzymes in the glycolytic and anaerobic fermentation pathways, allow a limited amount of ATP synthesis in an oxygen-independent manner (Sachs et al. 1996; Vartapetian and Jackson 1997). In previous anaerobic studies, Ala, Val, Gly, Leu, Arg, Tyr, Phe, Pro, 4-aminobutyrate, and succinic acid were reported to accumulate in anaerobic tissues of rice (Narsai et al. 2009; Sup et al. 1981; Reggiani et al. 1988). In addition, an basic amino acid carriers (BAC), is enriched under anaerobic conditions, which is accompanied by an increase in mitochondrial arginase, arginine and ornithine levels, consistent with the anaerobic effect of BAC (Taylor et al. 2010). These compounds promote mitochondrial arginine metabolism and the plant urea cycle during nitrate assimilation under anaerobic germination conditions in rice (Taylor et al. 2010). Putrescine synthesized from arginine is also more abundant during the anaerobic growth of rice (Reggiani et al. 1989), and its synthesis is also related to the ethylene-mediated enhancement in rice cell elongation (Lee and Chu 1992).

Due to the relatively low amount of ATP produced under anaerobic conditions, anaerobic germination (AG) is a very challenging process that is strictly regulated by transcriptional and metabolic changes. However, to date, few studies have integrated rice seed AG metabolism, GWAS and transcriptomic data to study the regulatory mechanism of the AG process. Most studies have only analyzed the transcriptome or metabolome or GWAS. Hsu and Tung (2017) conducted transcriptome analysis on the tissues of six rice varieties with different AG tolerances after 7 days of flooding treatment. Genes related to cell wall modification, fermentation, transcriptional regulation and hormone biosynthesis were upregulated, which triggered a series of downstream transcriptional regulators to promote appropriate metabolic processes and morphological adjustment to cope with flooding stress. Narsai et al. (2009) studied the time-dependent metabolic response of rice seeds under different oxygen environments by nontargeted gas chromatography‒mass spectrometry (GC‒MS). A total of 110 differentially abundant metabolites were detected, 13 of which were defined as anaerobic reactors (their levels increased under anaerobic conditions and decreased under aerobic conditions), including GABA (γ-aminobutyric acid), succinic acid, phenylalanine, Orn (Ornithine), coumaric acid, uric acid, glycolic acid, fumaric acid, nicotinic acid, glycerol-3-phosphate and inositol. Rohilla et al. (2020) investigated the genome-wide association studies (GWAS) using 50 K rice genic SNP chip across 94 deep-water rice genotypes collected from different flood-prone districts/villages of Assam, 20 significant genes were identified and found to be associated with AG-related traits.

In this study, we first conducted a dynamic genome-wide association study (GWAS) of 591 natural rice populations under anaerobic conditions, and a total of 317 SNPs were detected, which enriched the genetic basis related to anaerobic germination process. Extensive targeting and targeted metabolomic analysis were then used to identify key metabolites that respond strongly to anoxic stress, enriching the regulatory networks associated with anaerobic metabolism. The WGCNA analysis results of metabolomics and transcriptomics were combined to uncover important regulatory genes of key metabolites related to anaerobic germination and enrich the regulatory network of gene metabolism. To further improve the molecular mechanism of rice hypoxic germination by studying the function of genes related to the regulation of seed hypoxic germination, and to provide theoretical basis for the selection of rice varieties suitable for direct seeding. This study provides new insights into the regulation of AG metabolism in rice seeds, improves our understanding of the molecular basis of AG and lays a foundation for the improvement of AG tolerance in rice seeds.

## Results

### Phenotypic Analysis of Coleoptile Traits of 591 Rice Germplasm Under Anaerobic Germination

A total of 591 rice varieties were harvested in the late season of 2019. The length (CL), surface area (CSA), volume (CV) and diameter (CD) of the coleoptile growing in 2d, 3d and 4d under anaerobic conditions were measured (Additional file 13: Table S12). The coefficient of variation of population phenotype ranged from 11.11% (2019-AN4d, CD) to 100% (2019-AN2d, CL), indicating that coleoptile length (CL), surface area (CSA), volume (CV), and diameter (CD) had a wide range of phenotypic variation under anaerobic conditions, with abundant variation (Table 1). Interestingly, the coefficient of variation of all four traits decreased with the increase of anaerobic time, which may be due to the rapid elongation of the coleoptile of the anaerobic tolerant varieties in the early anaerobic treatment, thereby rapidly escaping from the hypoxic environment. In addition, all traits exhibited continuous unimodal distributions. The skewness and kurtosis absolute values of the phenotype values for traits such as embryo sheath length in AN4d, embryo sheath diameter in AN3d and AN4d were very close to 0.5 (Table 1 and Additional file 1: Fig. S1–S2), indicating that these traits closely approximate a normal distribution. Combined with the frequency distribution histogram, it can be seen that the value of each phenotypic trait basically conforms to normal and partial normal distribution, indicating that the genes controlling these phenotypic variations are multiple minor genes, and these traits are regulated by multiple genes, which conforms to the genetic law of quantitative traits. These results indicate that the phenotypic values of the coleoptile of rice seeds under anaerobic can be used for association analysis in order to excavate relevant loci in response to anaerobic germination.

**Table 1:** Statistical analysis of phenotypic traits of coleoptile of related populations during germination under submergence condition

Traits	Env	Mean ± SD	Range	Skewness	Kurtosis	CV (%)
CL (cm)	2019-AN2d	0.11 ± 0.1	0–0.72	2.01	5.78	90.91
	2019-AN3d	0.55 ± 0.43	0.02–2.55	1.31	1.96	78.18
	2019-AN4d	1.31 ± 0.64	0.07–3.19	0.45	− 0.38	48.85
CSA (cm^2^)	2019-AN2d	0.02 ± 0.02	0–0.12	2.47	8.12	100.00
	2019-AN3d	0.09 ± 0.07	0.01–0.45	1.47	2.71	77.78
	2019-AN4d	0.23 ± 0.12	0.01–0.58	0.62	− 0.17	52.17
CV (mm^3^)	2019-AN2d	0.27 ± 0.22	0–1.6	2.38	7.42	81.48
	2019-AN3d	1.17 ± 1	0–6.47	1.67	3.58	85.47
	2019-AN4d	3.14 ± 1.81	0.18–8.93	0.79	0.12	57.64
CD (mm)	2019-AN2d	0.45 ± 0.08	0.1–0.65	− 1.29	3.5	17.78
	2019-AN3d	0.51 ± 0.06	0.35–0.7	0.48	− 0.14	11.76
	2019-AN4d	0.54 ± 0.06	0.41–0.76	0.5	0.2	11.11

### Genetic Analysis of Population Structure

Based on 126,841 SNPs, an evolutionary tree (NJ tree) was constructed using p-distance method. It was found that these materials could be divided into two groups: indica rice and japonica rice, in which there were 465 indica rice and 126 japonica rice. In addition, further analysis of indica rice materials showed that indica rice materials could be divided into 4 subgroups, POP1-POP4, of which POP1 subgroup had 24 materials, POP2 subgroup had 129 materials, POP3 subgroup had 112 materials, and POP4 subgroup had 200 materials. The evolutionary tree analysis of 591 rice natural populations is detailed in Additional file 1: Fig. S5.

### LD Decay Distance Selection

Among 126,841 SNPs, 14,129 SNPs were selected by equidistance method to analyze the level of LD decay in *indica* rice subsets, *japonica* rice subsets and all materials. Among them, The nonlinear regression model of mean* r*^2^ of *indica* rice subpopulation, japonica rice subpopulation and all materials can be expressed as y = − 0.108ln(x) + 0.8245 (*R*^2^ = 0.9710), y = − 0.115ln(x) + 1.0807 (*R*^2^ = 0.9704) and y = , respectively − 0.075ln(x) + 0.7144 (*R*^2^ = 0.9169). When *r*^2^ decays to half of the maximum value, the corresponding decay distances are 45.47 kb, 155.95 kb and 117.06 kb, respectively. LD decay distance of japonica rice materials was close to that reported in the past, while LD decay rate of *indica* rice materials was faster than reported in the past (Mather et al. 2007; Huang et al. 2010; Lu et al. 2015; Li et al. 2019), the reason may be related to the existence of subpopulations in *indica* rice materials. Based on the above analysis, the LD attenuation distance of the test population material was finally taken as 117.06 kb (Additional file 1: Fig. S6).

### Genome-Wide Association Analysis of 591 Rice Germplasm

Using a mixed linear model (MLM) and population structure and relationship matrix (Q + K) as covariables, a dynamic genome-wide association analysis was performed on the phenotypic traits of CL, CSA, CV and CD of 591 rice germplasm related populations in 2d, 3d and 4d during anaerobic germination (Additional file 1: Figs. S1–S4). To excavate QTL sites associated with anaerobic germination of rice seeds. Under the significance threshold − log(P) > 5, a total of 640 significantly associated SNP loci were detected, of which 282 loci were repeatedly located.

Considering that the LD attenuation distance of this population was 117.06 kb, 640 significant sites were combined with a window of 234.12 kb, that is, the physical distance between significantly related SNPs was less than 234.12 kb, and the most significant associated site was used as the candidate site in this region. A total of 317 associated loci were obtained (Additional file 5: Table S4). We further analyzed the distribution of 317 associated loci on chromosomes. The results showed that these 317 associated loci were distributed across all 12 chromosomes, with the largest number on chromosome 1, followed by chromosome 11, where chromosome 5 was associated with the fewest genetic loci, with only 16 (Additional file 5: Table S4).

In addition, this study found that among the sites associated with multiple traits, more sites were simultaneously associated with coleoptile length, surface area, and volume, while no sites were detected that coleoptile diameter was simultaneously associated with other traits. The physical locations of the significant sites in this study were then compared with previous QTLs detected by linkage or association mapping. It can be found that QTL *qAG-9-2*, which is finely located to *OsTPP7* gene encoding trehalose 6-phosphate phosphatase, has been located many times in the localization results of this study. The localization interval is determined to be 12169328 bp-12474371 bp. Interval length of 305,043 bp, and then through the genome browser (https://www.ricedata.cn/gene/gbrowser.aspx) to search range of the gene, found within the range of 40 candidate genes, Finally, further screening was conducted in combination with the differential genes of the transcriptome in this study, and 8 genes were found to be differential genes, including *OsTPP7*.

A total of 317 significant SNP loci were identified by GWAS analysis of 591 rice varieties, and further screening showed that 27 loci were close to or even overlapped with the reported QTL physical locations (Table 2). Analysis of GWAS localization results showed that the significant SNP identified by associated CDAN2d traits (Chr1-9773247) overlapped with the reported localization interval (8.29–11.75 Mb) of the important QTL *qAGP1* for hypoxic germination (Liu et al. 2021a). Liu et al. (2021a) also found three important genes related to glycolysis and stress response during *qAGP1* interval analysis, namely pyruvate kinase (*LOC_Os01g16960*), peroxidase precursor (*LOC_Os01g16450*) and MYB transcription factor (*LOC_Os01g16810*), respectively, and they may also play an important role in anaerobic stress-related metabolic pathways or transcriptional regulatory networks. In this study, significant SNPs located at the 21775097 bp position on chromosome 12 by association of CVAN4d traits overlapped with two anaerobic germ-related *qCL-12* and *qCSA-12-1* regions located by Yang et al. (2019) using CL and CSA traits (21250000 bp–21900000 bp), and Yang et al. (2019) analyzed the transcriptome data of YZX and 02428 and found that the *Os12g0539751* screened from this interval showed highly variable expression levels at the transcriptome level. These results not only demonstrate the reliability of GWAS localization results, but also demonstrate that the combination of GWAS with metabolomic and transcriptomic studies is a powerful tool for mining novel candidate genes.

**Table 2:** The information of 27 loci of the 317 SNP loci overlapped with the reported loci

Traits	Treatment	Chromosome	Peak position (bp)	Peak value	known loci
CD	CDAN2d	1	9,773,247	5.3599	*qAGP1*, Liu et al. (2021a)
	CDAN2d	2	5,392,354	5.9180	*qAG2.1*, Kim et al. (2019)
	CDAN4d	9	21,404,841	5.8758	*qCD-9-3*, Yang et al. (2019)
CL	CLAN2d	12	26,942,207	5.1454	*qCSA-12-2*, Yang et al. (2019)
	CLAN2d	3	32,459,894	5.4106	*S3_32459722*, Su et al. (2021)
	CLAN3d	3	36,409,153	5.1926	*qSUR3-1Rc222-SCR-14,* Ghosal et al. (2019)
CSA	CSAAN2d	1	25,169,822	5.4387	*qAG1*, Jeong et al. (2020); *S1_25099585*, Su et al. (2021)
	CSAAN3d	2	26,714,756	6.5526	*qSSD-2*, Li et al. (2020); *qAG2*, Septiningsih et al. (2013)
CV	CVAN2d	1	31,418,900	6.7756	*qAG-1-2*, Angaji et al. (2010); *S1_31006962*, Hsu and Tung (2015)
	CVAN2d	1	38,223,398	8.6772	*qCLN1*, Liu et al. (2021a)
	CVAN2d	1	38,596,277	5.6269	*qCD-1*, Yang et al. (2019)
	CVAN2d	2	20,748,476	5.3130	*qGS2.1*, Chen et al. (2012); *qAG-2*, Angaji et al. (2008); *qAG-2-1*, Angaji et al. (2010)
	CVAN2d	3	23,491,435	5.0665	*qAG3-2,* Liu et al. (2021a, b)
	CVAN2d	6	9,925,796	11.7983	*qCSA-6* and *qCV-6-1*, Yang et al. (2019)
	CVAN2d	9	10,654,108	6.3812	*qAG9-3*, Liu et al. (2021b)
	CVAN2d	11	3,769,274	10.0400	*qAG11*, Jeong et al. (2020)
	CVAN3d	1	9,390,882	5.4285	*qAGP1,* Liu et al. (2021a)
	CVAN3d	3	8,956,500	5.2753	*qAGP3*, Liu et al. (2021a)
	CVAN3d	3	27,826,611	8.1694	*S3_27854371*, Su et al. (2021)
	CVAN3d	3	28,227,524	7.0063	*S3_28290634*, Su et al. (2021)
	CVAN3d	6	30,191,930	5.7992	*seq-rs3121*, Zhang et al. (2017)
	CVAN3d	10	12,877,840	5.0584	*qAG10-1*, Liu et al. (2021b)
	CVAN4d	1	9,494,163	5.1458	*qAGP1,* Liu et al. (2021a)
	CVAN4d	1	26,025,387	5.9391	*qAG-1-2,* Angaji et al. (2010)
	CVAN4d	7	4,973,098	5.2164	*seq-rs3210*, Zhang et al. (2017)
	CVAN4d	8	24,896,504	5.5620	*seq-rs3972*, Zhang et al. (2017)
	CVAN4d	12	21,775,097	5.8391	*qCL-12* and *qCSA-12-1*, Yang et al. (2019)

### Phenotypic Analysis of YZX and 02428 Seed Germination

**Fig. 1:**
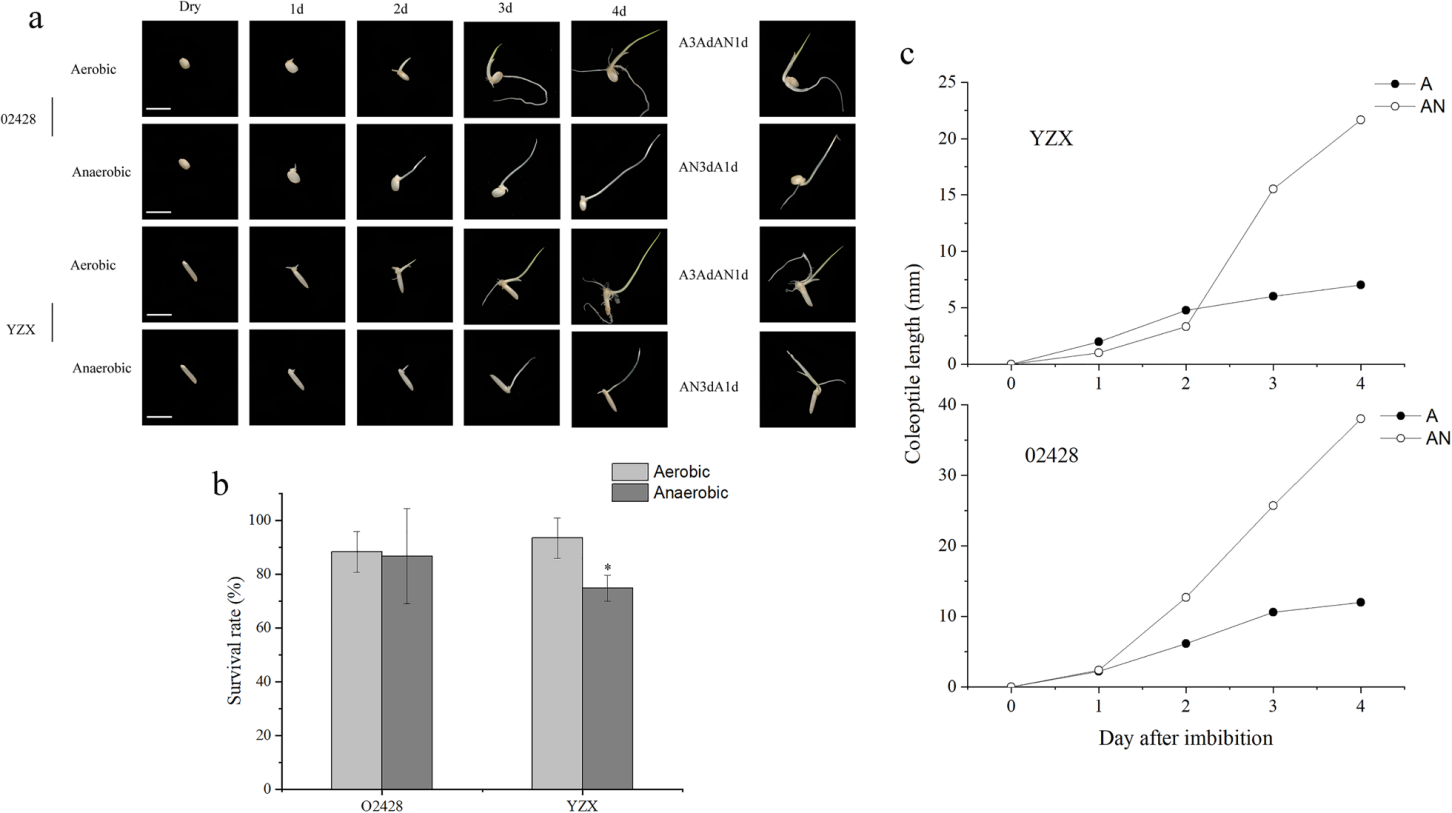
Germination of YZX and 02428 in aerobic and anaerobic environments at 30 °C. **a** germination of YZX and 02428 at 0–4 d. Simultaneously, two switching experiments were conducted: A3dAN1d, wherein seeds cultured in an aerobic environment for 3 days were transferred to a submerged environment for 1 day, and AN3dA1d, wherein seeds cultured in a submerged environment for 3 days were transferred to an aerobic environment for 1 day. Dry indicates dry seeds at zero days of germination. **b** survival rate of YZX and 02428 after aerobic and anaerobic 4 d treatment, c, YZX and 02428 in aerobic and anaerobic treatment of 0–4 d changes in the length of the coleoptile. *p < 0.05

In this study, two kinds of rice seeds (02428 for *Japonica* rice, YZX for *Indica* rice) were submerged (anaerobic, represented by AN) for 0, 2, 3, and 4 days or cultured aerobically (aerobic, represented by A; that is, not submerged). In order to gain greater insight into the regulatory processes that modulate responses to anaerobic conditions, (Howell et al. 2007), we employed two additional experimental designs to monitor the transition from aerobic to anaerobic (submerged treatment) states. At the same time, the experimental design switched the seeds of anaerobic germination to aerobic conditions, and samples were collected at the same time point. One was aerobic cultivation for 3 days followed by submergence (aerobic → anaerobic, represented by A → AN) for 1 day; the other was submergence for 3 days followed by aerobic cultivation (anaerobic → aerobic, represented by AN → A) for 1 day (Fig. 1a).

The seeds of YZX and 02428 germinated and developed coleoptiles and radicles under aerobic conditions, but the growth of the coleoptiles and radicles of both seeds was slower after switching from aerobic to anaerobic treatment (A3dAN1d) than under aerobic treatment (A4d). Under anaerobic conditions, both seeds developed coleoptiles without radicles, but the radicles were developed after switching from anaerobic to aerobic treatment (AN3dA1d). However, both seeds still only developed coleoptiles without radicles after 4 days of anaerobic (AN4d) treatment (Fig. 1a). The analysis of the germination rate of YZX and 02428 after 4 days of germination suggested that there was no significant difference in the germination rate of 02428 under aerobic and anaerobic conditions, while the germination rate of YZX after anaerobic treatment was significantly lower than that under aerobic treatment (Fig. 1b). In addition, the phenotypic analysis of the 0–4 d coleoptile length (CL) of YZX and 02428 showed that the CL of both seeds under anaerobic conditions was higher than that under aerobic conditions, and the CL of 02428 was significantly higher than that of YZX under anaerobic treatment (Fig. 1c), which indicated that 02428 might be more resistant to flooding.

### Changes in Metabolite Abundance of YZX and 02428 Under Aerobic and Anaerobic Germination

The Total Ions Current (TIC) plot represents a continuous description of the intensity sum of all ions in the mass spectrum at different time points (Additional file 1: Fig. S7). The Additional file 1: Fig. S8 shows an overlay of the TIC plots between the first and last QC samples, indicating that the TIC plots of the metabolites had a high degree of overlap. This result suggests that the Retention Time (RT) and peak intensities were consistent between the two QC samples, signifying good signal stability in the detection of the same sample at different times. Therefore, these results indicated that the data recorded in this study have good repeatability and reliability. The PCA scatter plots of all the samples (including QC samples) are shown in Additional file 1: Fig. S9. PCA principal component analysis clearly separated the two varieties and oxygen treatments, where the first principal component (pc1) separated the different oxygen treatment groups, and its contribution value reached 36.68%; the second principal component (pc2) separated the two varieties, and its contribution value reached 14.28%. This led us to further confirm that the different oxygen environments were the main factor affecting the anaerobic germination of these two rice varieties.

**Fig. 2:**
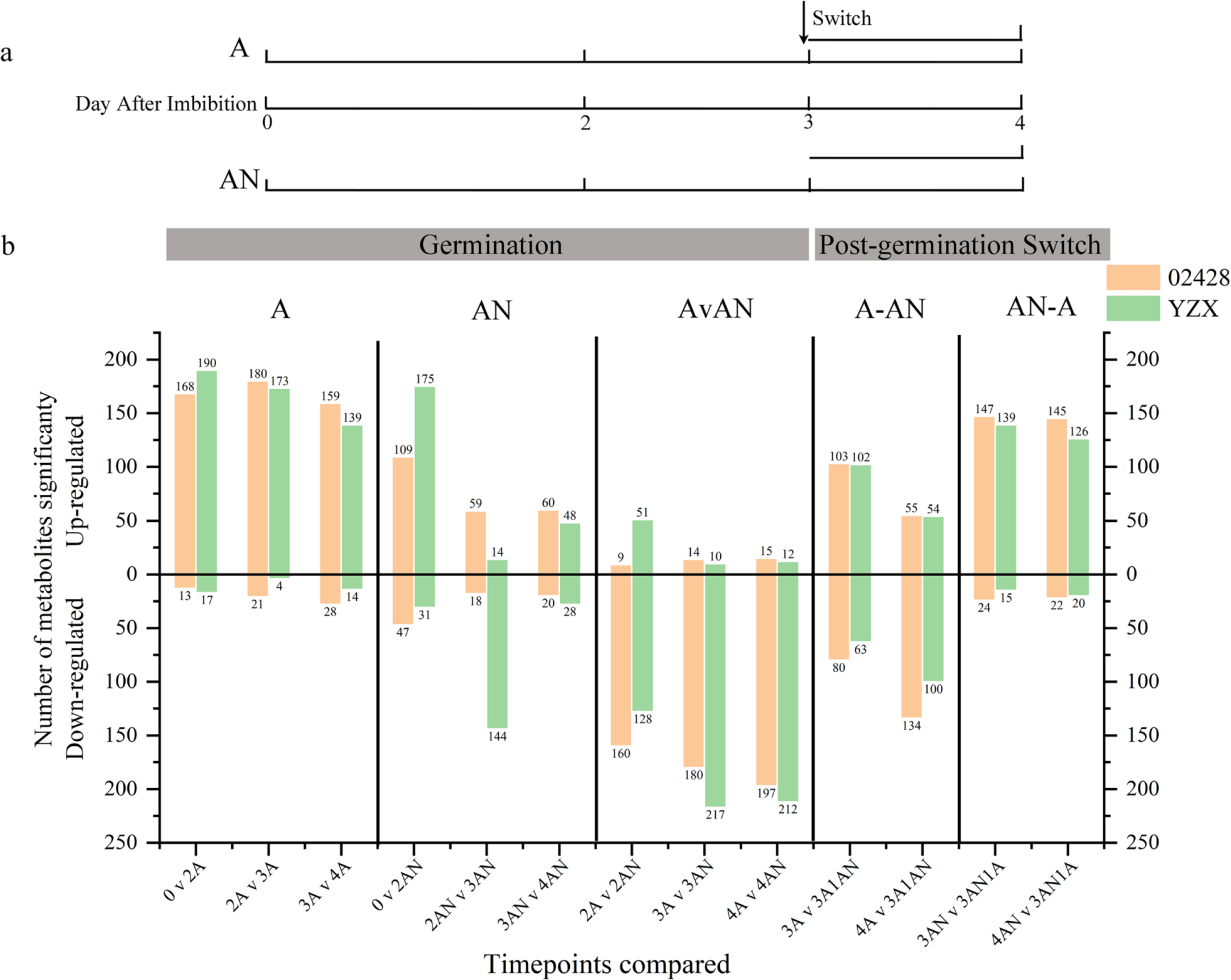
Analysis of metabolite abundance changes in two directly seeded rice varieties at different time points. **a** Overview of the experimental design. Seeds of two directly seeded rice varieties were imbibed in liquid medium under aerobic or submerged conditions. Three biological replicates for analysis of metabolism were obtained at 0, 2, 3, and 4 DAI (day after imbibition) under both growth conditions (the 0-d time point was common to both treatments). Additionally, after 3 days of growth, samples were switched, where aerobically grown samples were switched to anaerobic conditions, and samples were taken 1 d after switching. Submerged germinated seeds were switched to aerobic conditions, and samples were taken at the same time points. This yielded six datasets in which metabolic changes in metabolite abundance were analyzed. Time points for metabolite profiling analysis are indicated in gray. Three additional time points were included. Red indicates aerobic conditions, and blue indicates submerged conditions. **b** Summary of the changes in metabolite abundance during the experiments outlined in A. The number of metabolites is indicated on the ordinate. The time points compared are indicated on the abscissa

This study analyzed the abundance of 02428 and YZX metabolites in different oxygen treatment environments (Fig. 2b, Additional file 6: Table S5 and Additional file 7: Table S6). The study found that under aerobic (A) conditions, the two kinds of rice seeds had similar germination metabolic profiles, and the metabolites were mainly up-regulated, with no significant changes in abundance. Under anaerobic (AN) conditions, the abundances of metabolites produced by the germination of the two rice seeds were quite different. The abundance of metabolites changed significantly after germination after flooding for 2 days, the abundance of down-regulated metabolites in YZX increased sharply, this may be due to the decreased metabolism of YZX seeds after sensing hypoxia stress after 2d flooding, resulting in a decrease in up-regulated metabolites. The changes in metabolite abundance in 02428 were different from those in YZX, and the up-regulated metabolite abundances were all higher than the down-regulated metabolite abundances (Fig. 2b).

In the switching experiments, the down-regulated metabolite abundance increased when the seeds were switched from aerobic to anaerobic conditions. When switched from anaerobic to aerobic conditions, the seeds obtained sufficient oxygen, the metabolism was active, the abundance of up-regulated metabolites increased **(**Fig. 2b**)**, and the seeds grew radicles to obtain more oxygen and nutrients for seed growth (Fig. 1a).

A total of 730 metabolites were obtained based on dynamic widely targeted metabolomic technology combined with liquid chromatography-tandem mass spectrometry (LC–MS/MS). Firstly, statistics showed that 730 metabolites mainly included flavone (10.14%), organic acids (9.18%), amino acid derivatives (8.22%), nucleotide and its derivatives (7.81%), flavone C-glycosides (6.03%), hydroxycinnamoyl derivatives (4.79%), lipids_glycerophospholipids (4.66%), flavonol (4.52%) and amino acids (4.11%) (Additional file 1: Fig. S10i). Secondly, we analyzed the top three differentially expressed metabolites (DEM) with the highest proportion under aerobic and anaerobic. And we found that the up-regulated DEM were flavone (14.7%), nucleotide and its derivatives (7.71%) and flavone C-glycosides (7.47%) under aerobic. Different from aerobic, the up-regulated DEM under anaerobic were organic acids (10.57%), amino acid derivatives (10.19%) and amino acids (9.43%). In addition, the down-regulated DEM were flavone C-glycosides (13.89%), flavone (11.11%), amino acid derivatives (9.72%) and flavone (18.78%), flavonol (11.27%), flavone C-glycosides (10.80%) during aerobic and anaerobic, respectively (Additional file 1: Fig. S10 ii–v). Interestingly, we found that amino acids (4.1%) were only contained in up-regulated DEM, but not in down-regulated DEM under aerobic treatment. However, under anaerobic conditions, amino acids were contained both in up-regulated (9.43%) and down-regulated DEM (0.47%), but mainly in up-regulated DEM. And amino acids accounted for a larger proportion in up-regulated DEM under anaerobic when compared to aerobic (Additional file 1: Fig. S10 ii–vii).

### Screening of Metabolites Related to AG Tolerance in Rice Seeds by Weighted Gene Coexpression Network Analysis

**Fig. 3:**
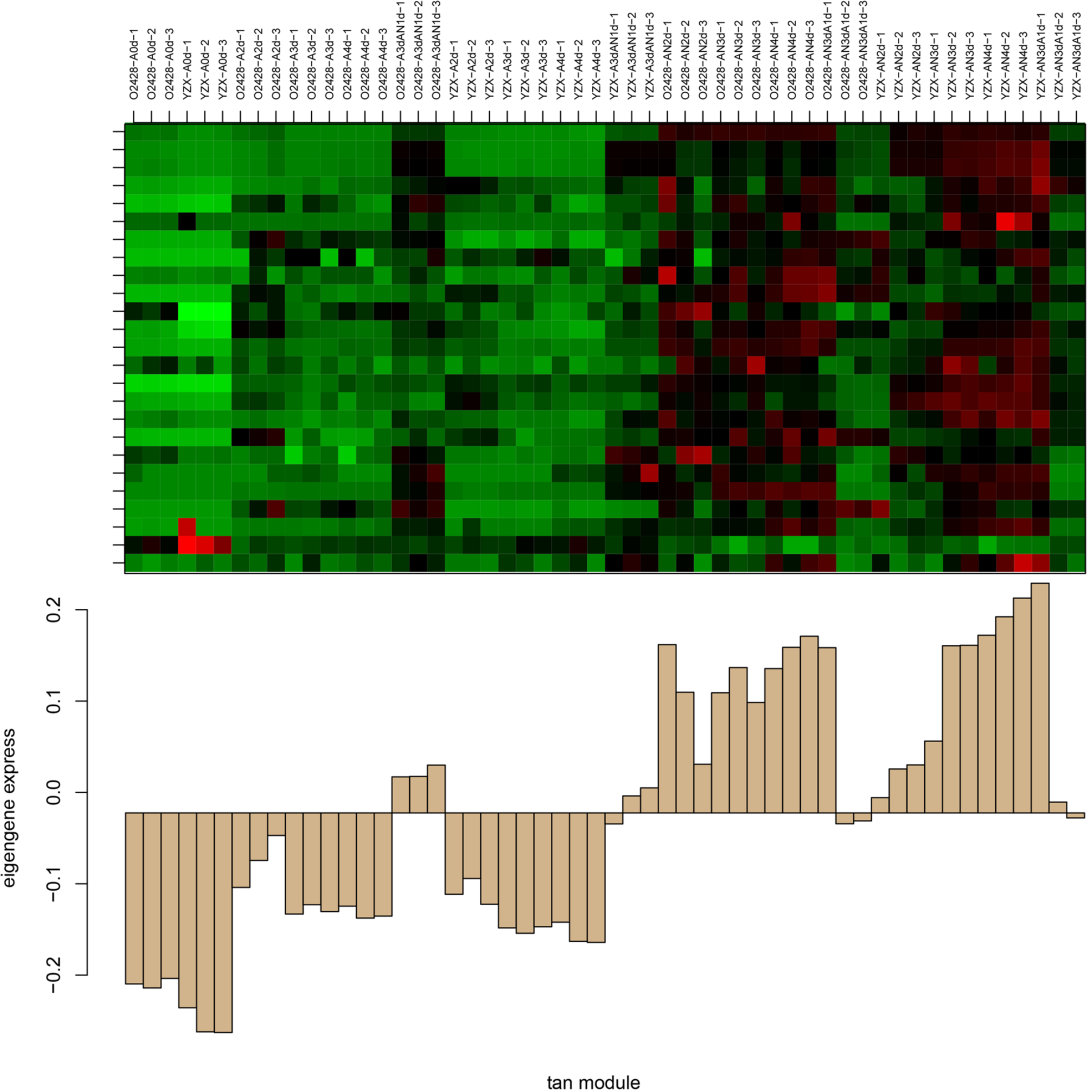
The expression patterns of metabolites in tan module

In this study, the WGCNA package tool was used to construct the coexpression module. We used flash clustering toolkit to cluster the samples and cluster genes with similar expression patterns to analyze the association between different modules and hypoxic germination-related phenotypes (Additional file 1: Fig. S11). In this study, when β = 9, the network was close to a scale-free network (Additional file 1: Fig. S12). Dynamic treecuts can identify modules in which metabolite expression values are very similar (Additional file 1: Fig. S13). After the highly similar modules were merged, a total of 11 modules were identified, ranging from 10 to 231 metabolites within the modules, with each module assigned a color as a reference (Additional file 8: Table S7). The expression pattern of the metabolites in each module in the sample is displayed with the module eigenvalues, and the sample expression pattern heatmap is shown (Additional file 1: Fig. S14). Through the heatmap of sample expression patterns, it was found that the metabolites in the tan module (Fig. 3) had low expression under aerobic conditions, increased expression under hypoxic conditions. Therefore, in this study, it is believed that the metabolite expression patterns in the tan module are consistent with the submergence-tolerant germination traits of the two rice varieties, and the metabolites in this module specifically respond to hypoxic stress (Fig. 3).

### Identification of Key Metabolites in Response to Flood-Tolerant Germination Traits in Rice Seeds

**Fig. 4:**
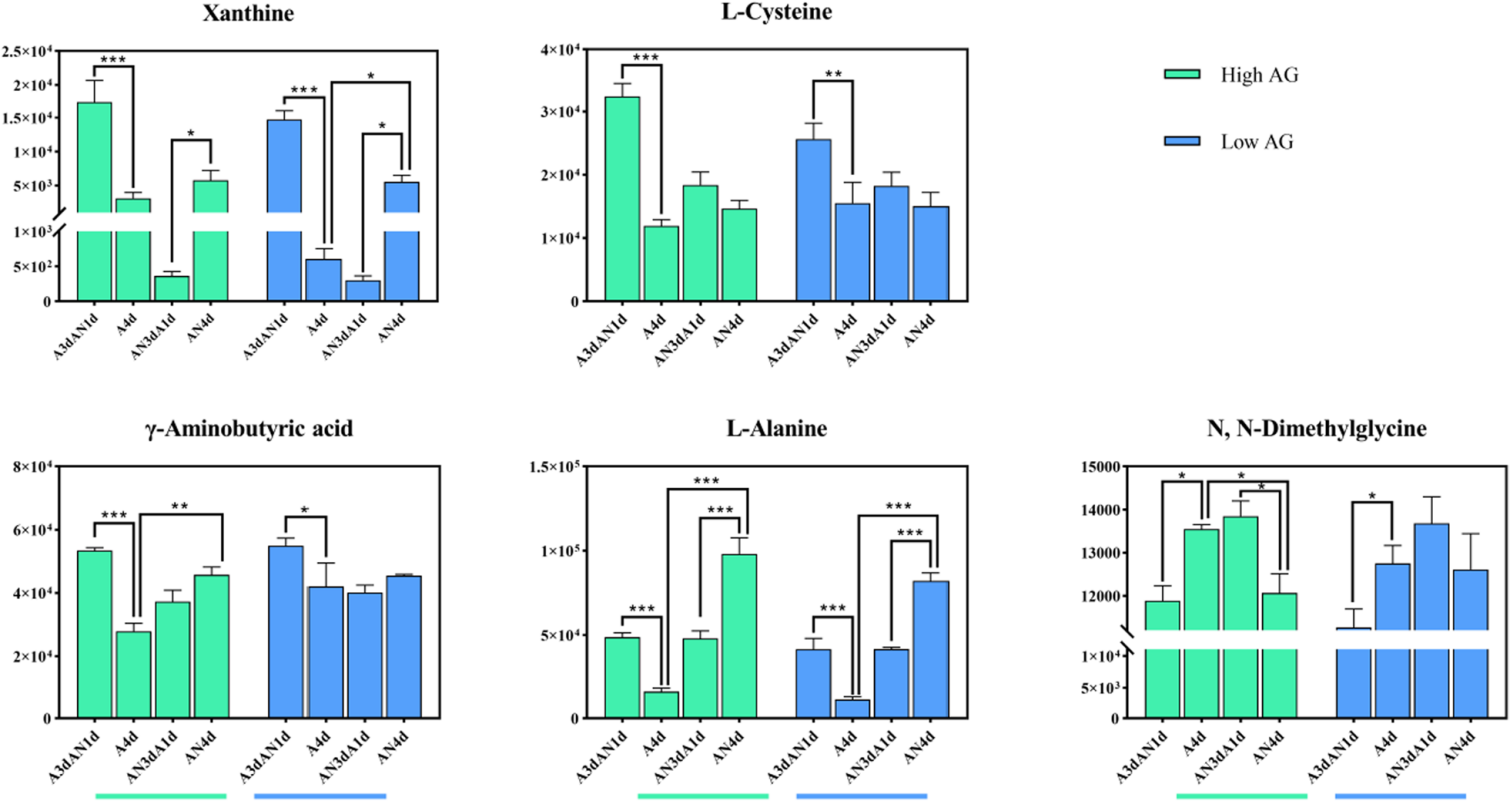
Levels of 5 metabolites in four kinds of High-AG (high germination ability under hypoxia) and four kinds of Low-AG (poor germination ability under hypoxia) rice germplasms

After comprehensively analyzing the fold change value (≥ 2 or ≤ 0.5), VIP value (≥ 1) and relative content of metabolites (≥ 10^6^) of each metabolite in the metabolite network diagram at the time points of A4d and A3dAN1d for the two materials 02428 and YZX in the tan module (Additional file 9: Table S8). Furthermore, to further verify and screen the core metabolites that are significantly related to the response of rice seeds to hypoxic stress, we determined the levels of these 5 metabolites in four High-AG (high hypoxia germination ability) and four Low-AG (low hypoxia germination ability) rice germplasms under the treatment of A4d, AN4d, A3dAN1d and AN3dA1d to identify and verify the core metabolites (Additional file 10: Table S9, Fig. 4).

Analysis of variance showed that the response of xanthine to hypoxic stress was more significant in the Low-AG germplasm, while γ-aminobutyric acid was more strongly responsive to hypoxic stress in the High-AG germplasm. l-alanine exhibited the most significant response to hypoxic stress in both the High-AG and Low-AG germplasms (*P* < 0.01). Its content was higher than that of the other substances, and it only significantly accumulated under hypoxic stress. Its content decreased immediately when it was transferred to aerobic conditions, and the High-AG germplasms were more tolerant than lower AG germplasms to hypoxia (*P* < 0.001): AN4d > A4d; AN4d > AN3dA1d; A4d < A3dAN1d.

Based on the accumulation and significance analysis of the metabolites, we hypothesize that xanthine, l-alanine and γ-aminobutyric acid may be sensitive and marker metabolites involved in the response to hypoxic stress perception.

### Genetic Basis for Dynamic Changes in Marker Metabolites During the Hypoxic Germination of Rice Seeds

To study the genetic basis of the dynamic changes in marker metabolites during the hypoxic germination of rice seeds, we performed hypoxic treatment (AN4d), aerobic treatment (A4d) and oxygen conversion treatment (AN3dA1d&A3dAN1d) on High-AG and Low-AG germplasms, and RNA-Seq data for 24 samples were generated. A total of 149.61 Gb of clean data was obtained. The average amount of clean data for each sample was 6.23 Gb, the average Q30 was 95%, and the average comparison rate was 93.3% (Additional file 11: Table S10). *r*^2^ indicates the correlation between biological replicates (Additional file 1: Fig. S15). A total of 15211 differentially expressed genes (DEGs) were detected in the two rice germplasms under different oxygen treatments (FPKM > 1, log (FC) > 1), and the PCA results and cluster dendrogram of the transcriptome (Additional file 1: Fig. S16) were obtained. The first principal component separated the aerobic treatment from the anaerobic treatment with an explanatory power of 55%, and the second principal component separated the High-AG and Low-AG germplasms. The AG-capable material was separated, with an explanatory power of 20.7%. This was the same expression pattern as that of the candidate marker metabolites, and overall, both the metabolome and transcriptome showed significant hypoxia response specificity.

To further understand the regulatory network of marker metabolite changes during the hypoxic germination of rice seeds, weighted gene coexpression network analysis (WGCNA) was performed to investigate the coexpression network of DEGs and marker metabolites. A total of 18 coexpression modules were identified based on their similar expression patterns (Additional file 1: Fig. S17, S18a and Additional file 12: Table S11). The module correlation heatmap (Additional file 1: Fig. S18b) showed that the content of l-alanine and xanthine were positively correlated with gene expression in the dark green (*P* = 1.0638e-9) and light green module (*P* = 5.0753e-11). The content of γ-aminobutyric acid was negatively correlated with gene expression in the dark gray module, with a coefficient of − 0.8059 (*P* = 2.00221e-6). These results suggest that the genes in these three modules are mainly involved in regulating the change in the l-alanine, xanthine, and γ-aminobutyric acid content during the process of rice seed germination under hypoxia.

**Fig. 5:**
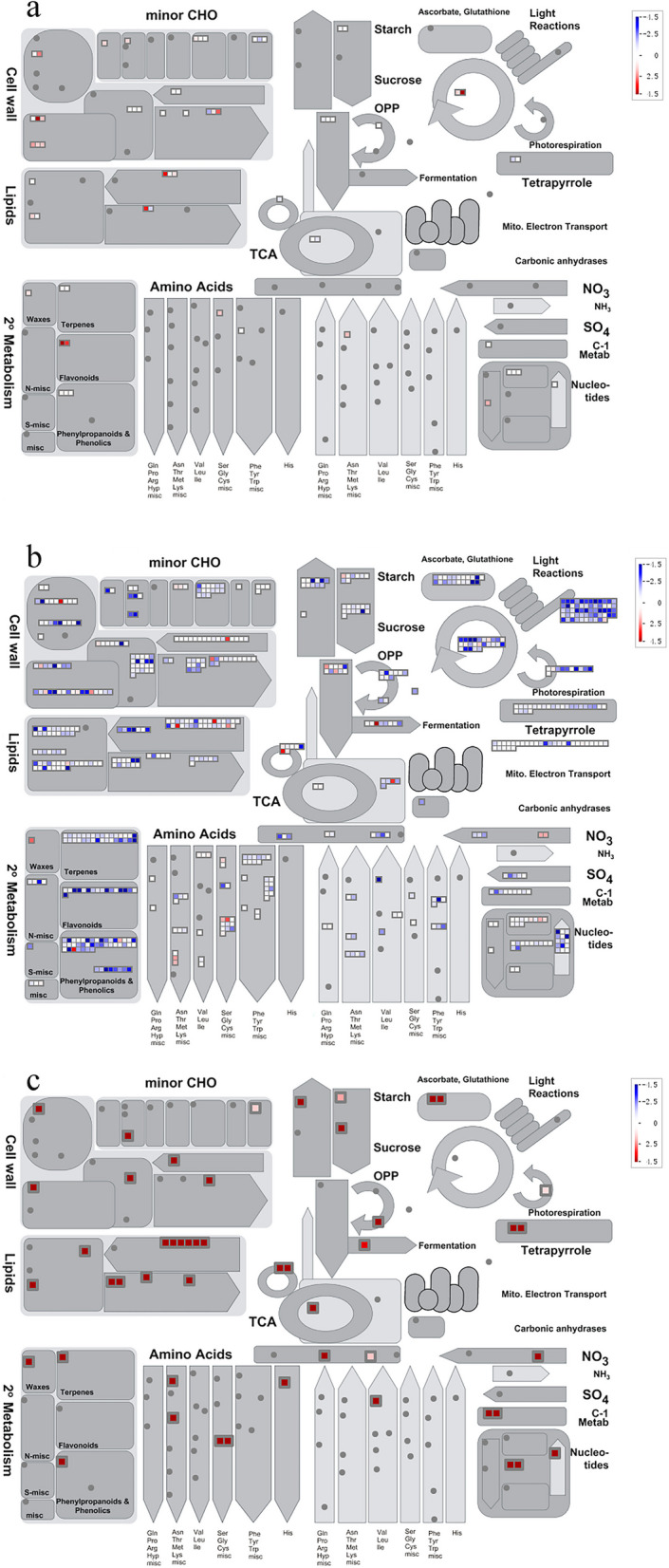
MapMan metabolic annotation map. **a** is the dark green module, **b** is the dark gray module, and **c** is the light green module

To further understand the functions of DEGs associated with marker metabolites during seed germination under flooding and coleoptile growth, the pathways involved and changes in genome-wide expression profiles were investigated. We performed GO and KEGG enrichment analyses on the genes in these three modules and used MapMan software to visualize the biological processes and metabolic pathways associated with the differentially expressed genes in the three modules, respectively (Fig. 5). For both types of enrichment analysis, FDR < 0.05 was used as the threshold. GO enrichment analysis showed (Additional file 1: Fig. S19) that genes in the dark green module were mainly associated with transporter activity, transmembrane transporter activity, molecular function regulator, enzyme function regulator, and cellular function in the molecular function category; plasma membrane part, anchored component of membrane and anchored component of plasma membrane in the cellular component category; and response to auxin in the biological process category. The genes of the light green module were mainly associated with transferase activity, purine ribonucleoside triphosphate binding, and ATP binding in the molecular function category, neuromuscular junction and synapse in the cellular component category, and response to inorganic substance and cellular modified amino acid biosynthesis in the biological process category. The genes of the dark gray module were mainly involved in catalytic activity in the molecular function category; cytoplasm, cytoplasmic part, and plastid in the cellular component category; and photosynthesis in the biological process category. KEGG enrichment analysis provides diverse insights into the molecular mechanism underlying seed germination under flooding and coleoptile growth processes.

### Involvement of l-alanine, GABA and Xanthine in the Regulatory Network of Hypoxic Germination

**Fig. 6:**
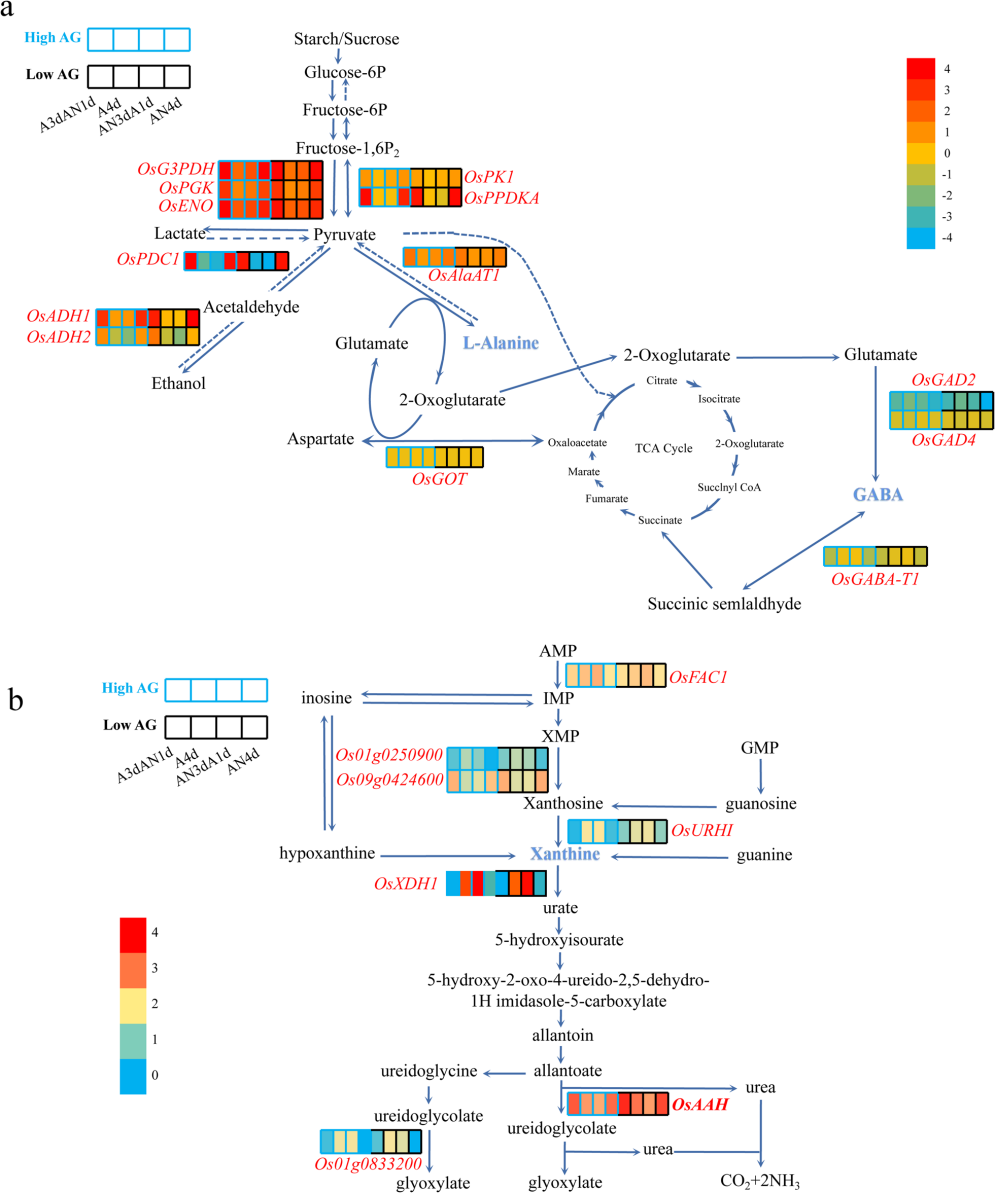
Identification of key structural genes in the metabolic regulatory network of hypoxic germination involving l-alanine, GABA and xanthine. **a** The metabolic regulatory network of hypoxic germination involving l-alanine and GABA and the structural genes involved in the dark green and dark gray modules. **b** Xanthine associated metabolic regulatory network of hypoxic germination and seven structural genes identified in DEGs

The accumulation of l-alanine and GABA is highly correlated with the dark green and dark gray modules. To generate the regulatory network associated with GABA, we examined all the DEGs as well as the dark green and dark gray modules. Structural genes were associated with the dark gray module. We identified 15 genes for l-alanine and GABA metabolism, including five alanine aminotransferase (*AlaAT*), one glutamate dehydrogenase (*GDH2*), five glutamate decarboxylase (*GAD*), three γ-aminobutyrate transaminase (*GABA-T*), and one aspartate transaminase (*GOT*) genes. Among them, only five DEGs were clustered in the dark green and dark gray modules (Fig. 6a), namely, *AlaAT1*, *GAD2*, *GAD4*, *GABA-T1*, and *GOT*, whose transcript levels were highly correlated with the levels of l-alanine and GABA. However, qRT‒PCR showed that *OsAlaAT1* (*Os10g0390500*) and *OsGAD4* (*Os03g0720300*) responded significantly to hypoxia treatment in both the High-AG material and Low-AG material, and their expression levels decreased once the material was transferred to aerobic treatment. The expression levels of *OsGAD2*, *OsGABA-T1* and *OsGOT* were higher under aerobic treatment but not specifically under hypoxia treatment (Additional file 1: Fig. S20). Based on genetic and biochemical evidence, we believe that *OsAlaAT1* and *OsGAD4* are the key structural genes that best characterize the metabolic process of l-alanine and GABA.

To date, the xanthine catabolism pathway during germination of rice under flooding is not clear. We used xanthine metabolism during seed germination in *Arabidopsis thaliana* as the reference and identified 7 structural genes among all the DEGs (Fig. 6b), namely, *Os05g0349200* (*OsFAC1*), *Os01g0250900*, Os03g0429800 (*OsXDH1*), *Os09g0424600*, *Os08g0557900* (*OsURHI*), *Os06g0665500* (*OsAAH*), and *Os01g0833200*, which are involved in the xanthine catabolic pathway. Among them, *Os09g0424600* and *Os06g0665500* (*OsAAH*) encode 5′-nucleotidase and allantoicase, allantoate amidohydrolase, respectively, which decompose purine nucleotides to synthesize xanthine and finally convert uric acid and allantoin into CO_2_ and NH_3_. The transcript levels are highly correlated with the level of xanthine and specifically respond to hypoxic stress. We consider *Os09g0424600* and *Os06g0665500* (*OsAAH*) to be good candidate genes for key enzymes of the xanthine catabolic pathway.

### Functional Exploration of *OsAlaAT1*

**Fig. 7:**
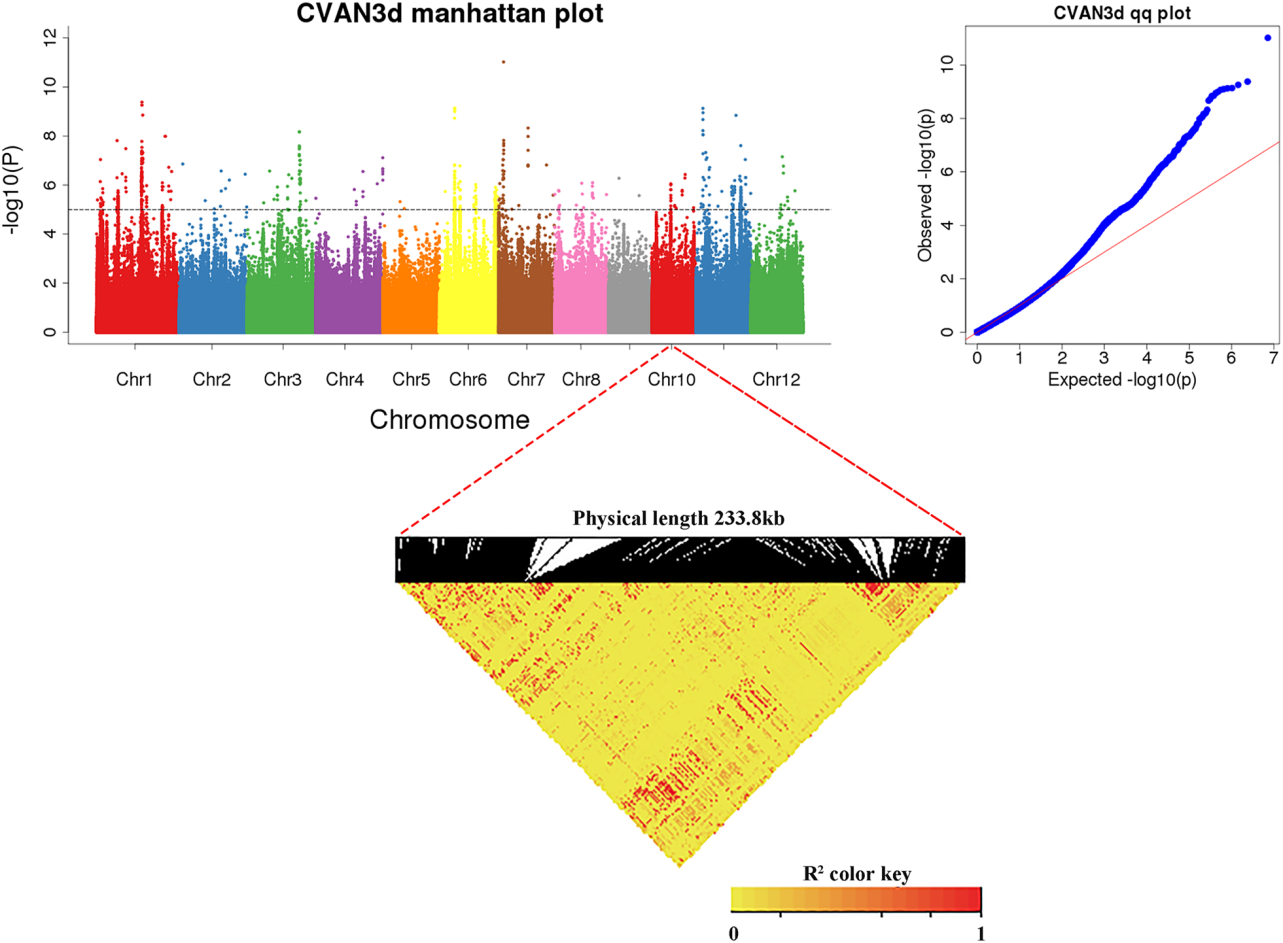
Genetic locus analysis of Chr10-12877840 in CVAN3d localization results

Under a significance threshold of − log(P) > 5, Chr10-12877840 was detected in the loci related to the CV trait (CVAN3d) after anaerobic treatment for 3 days. The results show that *OsAlaAT1* is located in Chr10-12877840. The LD heatmap shows a high correlation between 12936370–12994900, while the physical location of *OsAlaAT1* is 12967817–12974250, which is within the GWAS location range of CVAN3d (Fig. 7). Combined metabolome and transcriptome analysis showed that *OsAlaAT1* was a more reliable candidate gene.

**Fig. 8:**
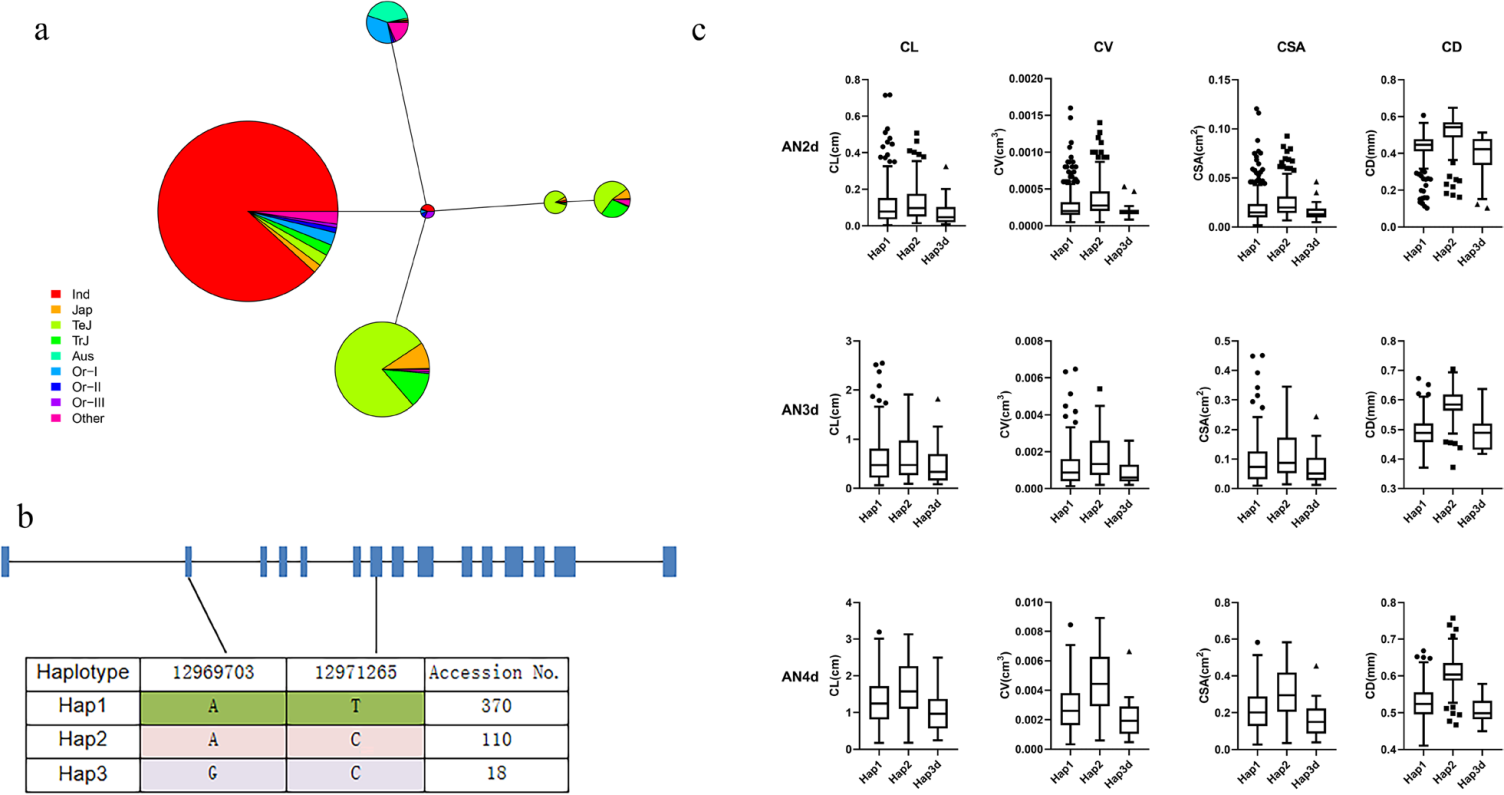
Haplotype analysis of *OsAlaAT1.* **a** haplotype network of *OsAlaAT1.* The size of circles is proportional to the number of samples for a given haplotype. IND indica, JAP japonica, TEJ temperate japonica, TRJ tropical japonica, Or Oryza rufipogon. **b** The three main haplotypes of *OsAlaAT1.* **c** Phenotypic values for 3 haplotypes of *OsAlaAT1*

To shed more light on breeding selection for *OsAlaAT1*, We use ECOGEMS (https://venyao.xyz/ECOGEMS/) and RFGB (https://www.rmbreeding.cn/Index/) for *OsAlaAT1* CDS are analyzed in haploid type, We identified three major haplotypes of LOC_Os10g25130 (RAP-DB id: Os10g0390500) (Fig. 8). Then, the proportion of the three haplotypes in 591 materials and the coleoptile-related phenotypes were analyzed. The results showed that the coleoptile length (CL), coleoptile surface area (CSA), coleoptile volume (CV) and coleoptile diameter (CD) of Hap2 in 2d, 3d and 4d were higher than those of Hap1 and Hap3, and Hap2 may be a better haplotype.

Phenotypic analysis was performed on the homozygous mutant strain *ko-alaat1* and the overexpression strain *pOX-OsAlaAT1* harvested from the T_2_ generation. The sequence alignment results showed that the homozygous mutation type of *ko-alaat1* was caused by the deletion of 1 bp base A at position 40 of the CDS region (483 amino acids) of *OsAlaAT1*, which resulted in the conversion of the 14th amino acid from Lys (K) to Arg (R). Translation was then terminated prematurely after encoding the 15th amino acid Phe (F), and this was a valid mutant with the protein sequence MAAPSVAVDNLNPRF*.

**Fig. 9:**
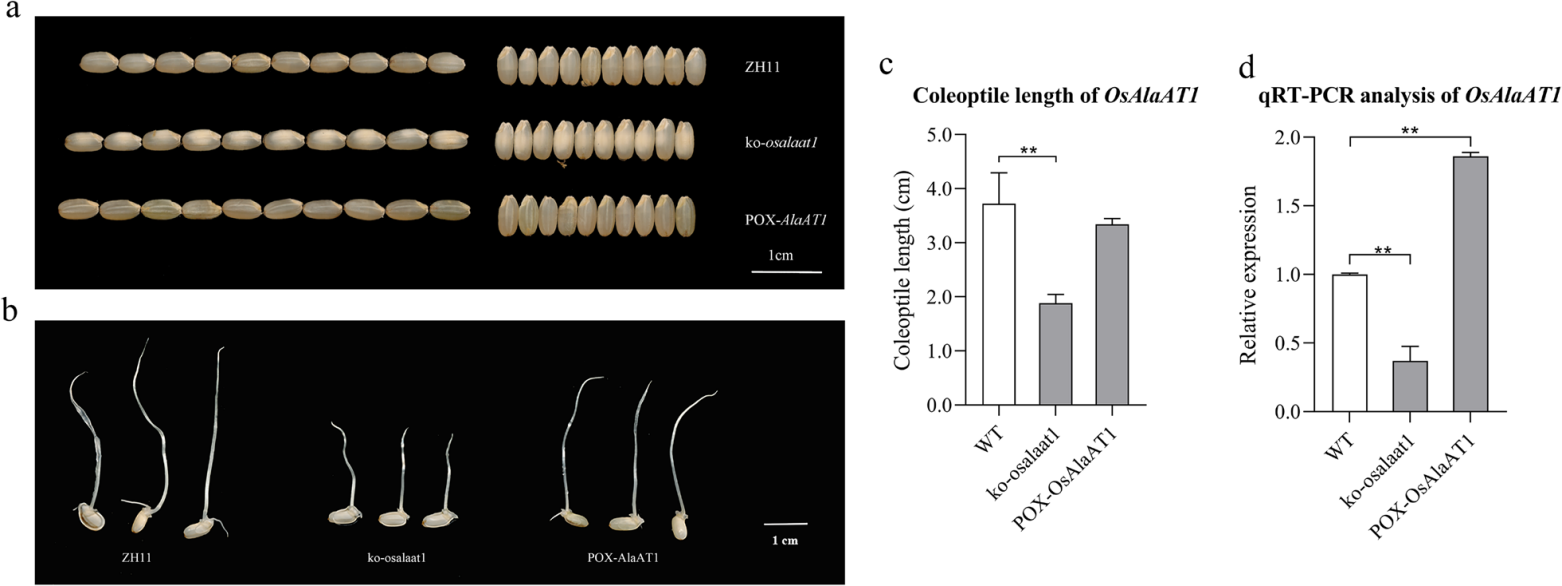
Phenotypic analysis of *OsAlaAT1* transgenic plants. **a** The phenotypes of *OsAlaAT1* transgenic plants in grain length and width. **b** and **c**, The phenotypic analysis of coleoptile length (CL) of *OsAlaAT1* transgenic plants under hypoxic conditions. **d** The qRT‒PCR results showed that the relative expression level of *OsAlaAT1* transgenic plants. **p < 0.01

**Fig. 10:**
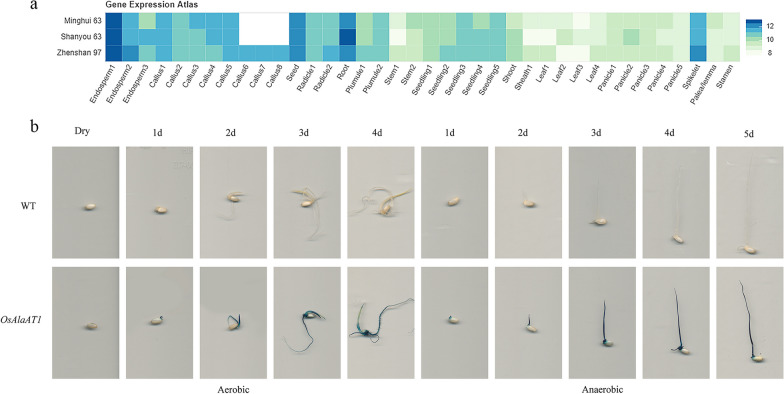
GUS staining analysis of *OsAlaAT1.* **a** Heatmap show the expression data of *OsAlaAT1* of three cultivars, Minghui63, Shanyou63, Zhenshan97, the color depth corresponding to expression level. **b** Expression pattern of GUS protein in germinating seeds

The phenotypes of *OsAlaAT1* transgenic plants showed no significant differences in grain length and width of *ko-alaat1* mutant and *pOX-OsAlaAT1* compared with the wild type (ZH11), but the chalkiness of the *ko-alaat1* mutant was more obvious than that of the control (Fig. 9a). In the analysis of the phenotypic results of coleoptile length (CL) of *OsAlaAT1* transgenic plants under hypoxic conditions, the CL of *pOX-OsAlaAT1* showed no obvious change, while the CL of *ko-alaat1* mutant was significantly reduced compared with that of the control (Fig. 9b-c). The expression profile of the CREP gene annotation (http://ricevarmap.ncpgr.cn/vars_in_gene/) showed that *OsAlaAT1* (*LOC_Os10g25130*) was highly expressed in the endosperm 5 days after pollination, germinated seeds, young roots and spikelets (Additional file 1: Fig. S21, Fig. 10a). The qRT‒PCR results showed that the relative expression level of the *ko-alaat1* mutant was significantly decreased compared with that of the control, which was consistent with the phenotype observation, while the relative expression level of *pOX-OsAlaAT1* was also significantly increased compared with that of the control, but the CL phenotype of *pOX-OsAlaAT1* was not significantly changed (Fig. 9b, d).

After 5 days of anaerobic treatment, GUS staining showed that *OsAlaAT1* was strongly expressed in the coleoptile, embryo, and radicle (Fig. 10b). This implies that *OsAlaAT1* might take part in seed AG treatment.

## Discussion

### A Total of 317 Significant SNPs Loci were Obtained by GWAS Analysis of 591 Rice Varieties

In this study, GWAS analysis was performed on CL, CD, CV and CSA of 591 natural rice varieties under 2d, 3d and 4d conditions of anaerobic germination, and a total of 317 related SNP loci were identified. Among them, the significant physical location of SNP located on chromosome 10 by using CVAN3d trait in this study is 12877840 bp. This single marker SNP was 61.985 kb away from the previously reported important QTL *qAG10-1* for hypoxic germination (Chr10-12815855) (Liu et al. 2021b). Significant SNP located in CLAN2d on chromosome 3 (Chr3-32459894) is only 172 bp apart from SNP identified in CDAN4d by Su et al. (2021) (Chr3-32459722). The peak SNP location on chromosome 1 was 31418900 bp, which was very close to another significant SNP (S1_31006962) reported to be associated with anaerobic germination (Hsu and Tung 2015). It also overlapped with the previously reported important QTL *qAG-1-2* for anaerobic germination, which was localized in 26-32 Mb (Angaji et al. 2010). In conclusion, after comparing with the reported literature, we found that 27 of the 317 SNP loci were selected to overlap with the reported QTL loci (Table 2), which further verified the reliability of the GWAS results in this study, and also proposed some newly discovered SNP loci related to hypoxic germination for reference. It provides the genetic basis for the screening of candidate genes related to anaerobic germination in the localization region.

### Amino Acid Metabolism and Sugar Metabolism Pathways are Crucial in the Anaerobic Germination of Rice Seeds

The metabolites accumulated in rice seeds during germination under hypoxic stress were mainly amino acids, amino acid derivatives, organic acids and their derivatives, nucleotides and their derivatives, phenolamine, and sugars (Additional file 1: Fig. S10). Amino acids and their derivatives are important nutrients in the process of seed germination and can be used to synthesize proteins required in the process of seed germination. Organic acids and their derivative secondary metabolites can participate in the TCA cycle and provide energy and raw materials for other metabolic processes. Phenolamines are a class of secondary metabolites that are widely distributed in the plant kingdom and play an important role in plant growth and development and the response to various biotic and abiotic stresses. Sugar is an important energy substance that can provide energy for the growth of germinating cells. The accumulation of these metabolites indicates that rice seeds have evolved corresponding regulatory strategies to resist submergence and hypoxic stress.

The sample expression pattern from the heatmap of the WGCNA results for metabolites showed that the expression of metabolites in the tan module was low under aerobic conditions, increased under flooding conditions, and decreased under aerobic conditions in response to hypoxic stress (Fig. 3). KEGG enrichment analysis of metabolites in the tan module showed that metabolites in this module were mostly enriched in pathways related to plant amino acid metabolism and sugar metabolism (Additional file 9: Table S8).

Activation of amino acid biosynthesis and recycling pathways is essential for seed germination. Amino acids are used not only for the synthesis of storage proteins but also as energy donors for the TCA cycle under plant development and stress conditions (Galili et al. 2014). For example, leucine (Leu), an essential branched-chain amino acid (BCAA), is an alternative energy source in mammals (Harper et al. 1984) and plants (Binder 2010). Leu promotes energy metabolism (glucose uptake, mitochondrial biogenesis and fatty acid oxidation) and improves protein synthesis (Duan et al. 2016). Likewise, the degradation of BCAAs provides energy for the early stages of Arabidopsis seed germination (Gipson et al. 2017). Sugar metabolism is important for plant adaptation to anaerobic environments (Adak and Saha 2021). Plants respond to hypoxic stress by switching from oxidative respiration to fermentative glycolysis to generate metabolic energy, which enables them to survive hypoxic stress (Tadege et al. 1999). The efficient utilization of sugar is essential for glycolysis and ATP production under hypoxic conditions (Mustroph et al. 2006). During rice seed germination and early seedling growth, the development of heterotrophic embryos is completely dependent on starch granules that are mainly stored in the endosperm of carbohydrates (Scofield et al. 2007). Kato et al. (Kato-Noguchi et al. 2011) also found that under anoxic conditions, the concentration of ATP in rice coleoptiles was higher than that in roots, and the concentration of sugar was also higher than that in roots under anoxic conditions. To adapt to the hypoxic environment caused by flooding, rice seeds mainly synthesize energy and metabolic raw materials required for life activities such as germination through amino acid metabolism and sugar metabolism pathways.

### Xanthine, L-alanine and GABA May be the Key Metabolites that are Highly Sensitive and Respond Strongly to Hypoxic Stress

Through extensive targeting and targeted metabolomic analysis, we identified three key biomarkers that were highly sensitive and responded strongly in response to perceived hypoxic stress. By combining this finding with transcriptomic data, we found a regulatory network that controls the production and accumulation of xanthine, l-alanine and GABA under hypoxic stress. By analyzing target metabolite levels (Additional file 10: Table S9, Fig. 4), amino acids (l-alanine, l-(-)-cystine, l-cysteine, l-serine) and nucleotides and their derivatives (2′-deoxyuridine, xanthine, substances of deoxyribose 5-phosphate, 5-methyluridine) were found to accumulate significantly in both the High-AG and Low-AG germplasms after the seeds were subjected to hypoxic stress, and their accumulation in the High-AG germplasms was higher than that in the Low-AG germplasms. The organic acid γ-aminobutyric acid has been reported to be involved in the stress response, growth and development, pest defense and other important life activities of plants (Zhang et al. 2020). Its level also increased when the seeds were under hypoxic stress, but the content of γ-aminobutyric acid in the High-AG germplasm was similar to that in the Low-AG germplasm. While the amino acid derivative *N*,*N*-dimethylglycine showed little difference in accumulation in the High-AG and Low-AG materials, the accumulation under aerobic treatment conditions was higher than that under hypoxic treatment conditions. Fructose 6-phosphate and glucose 6-phosphate, which are involved in the metabolic pathway of glycolysis, did not respond significantly when rice seeds were subjected to hypoxic stress, which may be related to their accumulation after starch hydrolysis under aerobic conditions being more strongly affected. Based on the accumulation and significance analysis of the metabolites, we hypothesize that xanthine, l-alanine and γ-aminobutyric acid may be more sensitive and strong marker metabolites in response to hypoxic stress perception. AG materials responded more strongly to hypoxic stress; the organic acid γ-aminobutyric acid responded more strongly to low oxygen stress in the High-AG material; the amino acid l-alanine showed a stronger response in both the High-AG and Low-AG material accumulation and responsiveness.

### Screening of Key Genes in Response to Hypoxia Specific Germination

Based on the combined analysis of the transcriptome and metabolites (Additional file 1: Fig. S17), we identified three modules that were highly correlated with xanthine, l-alanine, and γ-aminobutyric acid, namely, the dark green and light green modules (Additional file 1: Fig. S18). Through GO and KEGG analyses of the biological processes and metabolic pathways involved in the DEGs in the three modules (Additional file 1: Fig. S19), it was found that more than 60% of the genes in the three modules were involved in the pathways of carbohydrate metabolism, lipid metabolism, amino acid metabolism, and energy metabolism. Genes of the dark green and light green modules were upregulated in almost all metabolic pathways, especially cell wall metabolism, lipid metabolism, amino acid metabolism, starch and sucrose metabolism, upregulation of glycolysis, the TCA cycle, and flavonoid and nucleotide metabolism pathways. The genes of the dark gray module were downregulated in most metabolic pathways, but the starch and sucrose metabolism, glycolysis, TCA cycle, and amino acid metabolism pathways were upregulated.

In the regulatory network associated with l-alanine and γ-aminobutyric acid (GABA) (Fig. 6a), five DEGs were identified that were highly correlated with the levels of L-alanine and GABA. qRT‒PCR showed that *OsAlaAT1* (*Os10g0390500*) and *OsGAD4* (*Os03g0720300*) responded significantly to hypoxia treatment in both the High-AG material and Low-AG material and were the key genes that best characterized the metabolic processes of L-alanine and GABA. At the same time, the *Os09g0424600* and *Os06g0665500* transcript levels were highly correlated with the content of xanthine in the xanthine catabolism and catabolism pathway, and at the same time, they specifically responded to hypoxic stress and were good candidate genes for the key enzymes of the xanthine catabolism pathway (Fig. 6b). The purine base xanthine is a well-defined central intermediate in the catabolism of purine nucleotides in plants and can be degraded to urea by uric acid and allantoin and then converted to CO_2_ and NH_3_, releasing tetracyclic nitrogen as ammonia for reassimilation of amino acids. In addition, its catabolic intermediates uric acid, allantoin and allantoic acid can quench the harmful effects of reactive oxygen species (ROS) (Hofmann 2016), thereby promoting seed germination (Bailly et al. 2008).

### Preliminary Identification of the Anaerobic Phenotype of *OsAlaAT1*

During anaerobic metabolism, limiting lactate accumulation and maintaining cytoplasmic pH homeostasis is one of the strategies to confer hypoxia tolerance. Rice coleoptiles are able to grow in extremely low oxygen conditions. Anoxic stress increased the concentration of total free amino acids in the coleoptiles. Alanine (Ala) and γ-aminobutyric acid (GABA) are the main accumulated amino acids (Kato-Noguchi and Ohashi 2006). In hypoxic tissues, the alanine synthesis pathway competes with the lactic acid fermentation pathway for pyruvate, accompanied by an increase in gamma-aminobutyric acid (GABA), two amino acids that counteract the acidic effect of lactic acid. Furthermore, alanine synthesis protects the C3 backbone, thereby preventing a shortage of carbon availability and limiting the accumulation of the toxic compound acetaldehyde (Ricoult et al. 2006). In this study, WGCNA analysis of metabolomics and transcriptomics indicated that *OsAlaAT1*, *OsGAD4*, *OsAAH* and *Os09g0424600* may be important candidate genes affecting their metabolic processes. And *OsAlaAT1*, which is located in the GWAS localization range of CVAN3d, is considered to be a more reliable candidate gene. *OsAlaAT1*, as a key gene in the specific response to anaerobic conditions in High- and Low-AG materials during L-alanine metabolism, is of great research significance for the study of anaerobic germination in rice.

Through analysis of the phenotype of anaerobic coleoptile length (CL) of *OsAlaAT1* transgenic plants, it was found that although the anaerobic CL of *pOX-OsAlaAT1* did not change significantly, the CL of *ko-alaat1* mutant was significantly lower than that of the control (Fig. 9b–c), and the qRT‒PCR results also showed that the relative expression of the *ko-alaat1* mutant was significantly decreased compared with that of the control. Meanwhile, GUS staining showed that *OsAlaAT1* was strongly expressed in the coleoptile, embryo and radicle during seed germination under anaerobic treatment (Fig. 9b).

Using our dataset, we identified 3 marker metabolites, xanthine, l-alanine, and GABA, and 4 structural genes involved in their metabolic regulatory networks, namely, *OsAlaAT1*, *OsGAD4*, *Os09g0424600* and *Os06g0665500* (*OsAAH*). Our metabolic and transcriptomic datasets can be further used to identify key structural and regulatory genes involved in the relevant metabolism of rice seeds during hypoxic germination. This not only helps to refine the metabolic regulatory network controlling the hypoxic germination of rice seeds but also opens up new avenues for future breeding strategies aimed at improving rice seed quality.

## Materials and Methods

### Plant Materials and Treatment Conditions

In this study, seeds of two rice varieties were used for dynamic widely targeted metabolite assays: the *Indica* variety YZX and the *Japonica* variety 02428. YZX and 02428 were bred by the Hunan Academy of Agricultural Sciences and Jiangsu Academy of Agricultural Sciences, respectively. The research materials used in the dynamic targeted metabolomic analysis were AG-resistant materials (3, G539, G544, G546) and AG-sensitive materials (20, 220, G619, G628). The research materials for the dynamic transcriptomic analysis were AG-resistant materials (12, 55, 60, 126, 167, 258, 262, 264, 267, 268, 279, 282) and AG-sensitive materials (R404, R176, R98, R103, R198, R460, R462, R471, R111, R489, R509, R525). For specific material information, please refer to Additional file 2: Table S1. Detailed measurements are provided in the Additional file 14.

### Dynamic Widely Targeted and Targeted Metabolite Detection and Data Analysis

Metabolite profiling was carried out using a widely targeted metabolomic and targeted metabolomics method by Wuhan Metware Biotechnology Co., Ltd. (Wuhan, China) (http://www.metware.cn/). Reagents and methods for extracting metabolites were all carried out as outlined by Yan et al. (2019). Detailed measurements and data analysis for dynamic widely targeted metabolomic are provided in the Additional file 14. The methods (LC–MS/MS) for dynamic targeted metabolomics are as follows: a.) Sample pretreatment process: “A 50 ± 1.0 mg sample was added to 600 μL of 70% methanol aqueous solution, shaken for 10 min, and allowed to stand for 30 min. The operation was repeated 6 times. After standing in a refrigerator at 4 °C overnight, the sample was centrifuged at 4 °C and 12,000 r/min for 10 min, and 120 μL was taken.” b.) The analytical methods of LC–MS/MS: “The conditions for liquid chromatography spectrometry were as follows: the chromatographic column was an ACQUITY HSS T3 column (2.1 × 100 mm, 1.8 μm); mobile phase A was ultrapure water (containing 0.04% acetic acid); mobile phase B as acetonitrile (containing 0.04% acetic acid); the injection volume was 2 μL; And the column temperature was 40 °C.” c.) The mass spectrometry conditions: ion mode (ESI ±), curtain gas (35), ionSpray voltage (5500/− 4500), temperature (550), ion source gas 1 (50), ion source gas 2 (60), collision gas (medium), scan type (MRM), entrance potential (10/− 10), and collision cell exit potential (12/− 12).”

### Transcriptomic Sequencing

Total RNA extraction and purification were performed using Trizol™ reagent (Thermo Fisher) and the Plant Total RNA purification kit (Comwin Biotechnology Company), respectively. PCR amplification was performed to complete the preparation of the whole cDNA library. The cDNA library was sequenced on an Illumina sequencing platform (Illumina HiSeq™ 2500) by Gene Denovo Co., Ltd. (Guangzhou, China) (Su et al. 2021). Primers used for qRT-PCR were conducted as described in the Additional file 3: Table S2.

Differential expression analysis of two conditions/groups was performed using DESeq2 (Love et al. 2014). Genes with an adjusted *P* value < 0.01 found by DESeq2 were considered differentially expressed.

MapMan software (ver. 3.6.0RC1) (Usadel. 2005) was used to map transcriptomic data, define functional categories, and perform time-course analyses to identify significantly overrepresented functional groups. Osa_RAPDB_ mapping files were downloaded from the MapMan store server (http://mapman.gabipd.org/web/guest/mapmanstore).

### Weighted Gene Coexpression Network Analysis and Core Metabolite Selection

Coexpression networks were constructed using the WGCNA (v1.47) package in R (Langfelder and Horvath 2008). First, we performed a cluster analysis of these samples using the flash Clust toolkit (Additional file 1: Fig. S11). Soft threshold is one of the most critical parameters, which mainly affects the independence and average connectivity of coexpression modules. In order to determine the optimal value of the soft threshold and make the adjacency function satisfy the scale-free condition well, the logarithm of the number of connected nodes (log(i)) is negatively correlated with the logarithm of the probability of occurrence of this node (log(p(i))), and the minimum value when the correlation coefficient reaches the plateau period (or is greater than 0.8) is used as the soft threshold β for subsequent analysis. In this study, when β = 9, the network approaches scale-free network (Additional file 1: Fig. S12). Dynamic tree Cut (Additional file 1: Fig. S13) is a module divided according to clustering results, and the metabolite expression values in the modules are very similar. After highly similar modules were merged, each module was assigned a color as a reference. Meanwhile, the expression patterns of metabolites in each module in each sample were displayed with module eigenvalues, and the heat map of sample expression patterns was drawn (Fig. 3, Additional file 1: Fig. S14). Coexpression modules were constructed using the automatic network construction function blockwise modules with default settings. The networks were visualized using Cytoscape 3.3.7.

### Genome-Wide Association Analysis (GWAS)

Using a mixed linear model (MLM) and population structure and phylogenetic matrix (Q + K) as covariables, we conducted dynamic genome-wide association analysis the CL, CSA, CV and CD phenotypic traits in 591 rice germplasm associated populations harvested in the late cropping season in 2019 at 2 d, 3 d and 4 d under hypoxic conditions (Additional file 4: Table S3). Detailed methods are provided in the Additional file 14.

### Construction of *OsAlaAT1* Transgenic Vector and Acquisition of Transgenic Materials

To generate *ko-OsAlaAT1* mutants, small guide RNAs (sgRNAs) targeting *OsAlaAT1* were ligated into the expression vector pRGEB32. To create overexpression constructs, the coding sequences of *OsAlaAT1* were amplified by polymerase chain reaction (PCR) and cloned into pOX (overexpression vector), after which the *pOX-OsAlaAT1* construct was transferred into ZH11. Using the DNA of the ZH11 plant as a template, a promoter sequence 2 kb upstream of the *OsAlaAT1* coding region (ATG) was amplified by PCR and cloned into the pCAMBIA1305.1 vector to obtain the structure Pro_*OsAlaAT1*_:: GUS. Hygromycin was used as a selection marker and the constructed vectors were transferred into ZH11. Transgenic plants were obtained by agrobacterium-mediated rapid transformation method. The transformation was completed by Wuhan Biorun Biosciences Co., Ltd. The T_0_ generation positive plants were further used to obtain the T_2_ generation for preliminary phenotypic observation and analysis. The relevant primers are shown in Additional file 3: Table S2.

### GUS Staining

The GUS staining solution was prepared as follows: 50 mL 0.1 M PBS (pH = 7.0), 10 mL K-Fe-CN, 0.1 mL Triton-x-100 (Trilatone), 20 mL methanol, and 5 mL x-Gluc were dissolved in ddH2O, and the volume was fixed at 100 mL. After 7 days of waterlogging, the seeds were thoroughly immersed in GUS staining solution and reacted at 37 °C for 24 h. For tissue with deep background color, decolorization was necessary. The plant tissue was cleaned once with distilled water and decolorized with 75% ethanol at 50 °C until the chlorophyll completely faded.

### Statistical Analysis

Data were analyzed as described in the Additional file 14.

### Supplementary Information

**Additional file 1**. **Fig. S1.** Frequency distribution and Manhattan map and quantile-quantile (QQ) plots of SNPs associated with coleoptile diameter(CD) at different time points under anaerobic germination. **Fig. S2.** Frequency distribution and Manhattan map and quantile-quantile (QQ) plots of SNPs associated with coleoptile length(CL) at different time points under anaerobic germination. **Fig. S3.** Frequency distribution and Manhattan map and quantile-quantile (QQ) plots of SNPs associated with coleoptile surface area(CSA) at different time points under anaerobic germination. **Fig. S4.** Frequency distribution and Manhattan map and quantile-quantile (QQ) plots of SNPs associated with coleoptile volum(CV) at different time points under anaerobic germination. **Fig. S5.** Evolutionary tree analyses of 591 rice natural populations structure. **Fig. S6.** Estimation of genome-wide average LD decay distance. **Fig. S7.** Total ion current of one quality control sample by mass spectrometry detection (a) and multi-peak detection plot of metabolites in the multiple reaction monitoring mode (b). **Fig. S8.** Overlapping total ion flow chart (TIC chart) for QC sample mass spectrometry detection. **Fig. S9.** Principal component analysis (PCA) result. **Fig. S10.** Categorization of metabolites detected. **Fig. S11.** Sample Hierarchical Clustering Tree. **Fig. S12.** Power value curve. Analysis of network topology for various soft thresholding power. **Fig. S13.** Hierarchical cluster tree. **Fig. S14.** The expression patterns of metabolites in each module. **Fig. S15.** Correlation heatmap between samples. **Fig. S16.** Principal component analysis (PCA) result. **Fig. S17.** Eighteen modules heatmap of gene expression patterns. **Fig. S18.** Module partitioning and metabolite correlation heatmaps. **Fig. S19.** GO and KEGG enrichment analysis of dark green, dark gray, and light green modules. **Fig. S20.** qRT‒PCR results of OsAlaAT1, OsGAD4, Os09g0424600 and Os06g0665500 (OsAAH). **Fig. S21.** The spatio-temporal expression of LOC_Os10g25130 in Nipponbare based on RAP-DB and BAR database.

**Additional file 2**.** Table S1.** Rice Variety Source Information

**Additional file 3**. **Table S2.** Primers used for qRT-PCR and detection of OsAlaAT1 transgenic plants.

**Additional file 4**. **Table S3.** 591 rice germplasm source information.

**Additional file 5**. **Table S4.** 317 significant loci were identified by genome-wide association analysis of 591 rice germplasm.

**Additional file 6**. **Table S5.** Detected metabolites.

**Additional file 7**. **Table S6.** Classification of 730 metabolites.

**Additional file 8**. **Table S7.** Number of metabolites in 11 modules.

**Additional file 9**. **Table S8.** The VIP value, Fold Change value and Connectivity value of 25 metabolites in tan module under treatment with aerobic and then anaerobic conditions.

**Additional file 10**. **Table S9.** Contents of 5 metabolites in four kinds of High AG (high germination ability under hypoxia) and four kinds of Low AG (poor germination ability under hypoxia) rice germplasm.

**Additional file 11**. **Table S10.** Summary statistics for transcriptome sequencing quality and high qulity clean reads mapped to the reference genome.

**Additional file 12**. **Table S11.** Genes or metabolites within 19 modules.

**Additional file 13**. **Table S12.** Phenotypic data of CD, CL, CV and CSA.

**Additional file 14**. **Supporting Information. **Supplementary materials and methods.

## Data Availability

All the RNA-seq data generated in this research were deposited in the Sequence Read Archive database (www.ncbi.nlm.nih.gov/sra) at NCBI (National Center for Biotechnology Information) under accession number SRP282222. The datasets supporting the results of this study are included in the manuscript. Rice seeds are available from the National Engineering Research Center of Plant Space Breeding, PR China.

## References

[CR1] Adak MK, Saha I (2021). An updated overview of the physiological and molecular responses of rice to anoxia. Front Biosci.

[CR2] Angaji SA, Septiningsih EM, Mackill DJ, Ismail AM (2010). QTLs associated with tolerance of flooding during germination in rice (*Oryza sativa* L.). Euphytica.

[CR3] Bailly C, Bouteau HEM, Corbineau F (2008) Rôle de la signalisation par les espèces réactives de l'oxygène dans la germination et la levée de dormance des semences. Journal de la Société de Biologie: 241–248 10.1051/jbio:2008025 18980746

[CR4] Binder S (2010) Branched-Chain Amino Acid Metabolism in Arabidopsis thaliana. The Arabidopsis Book: e137 10.1199/tab.0137 PMC3244963 22303262

[CR5] Chen SL, Wang JM, Pan YZ, Ma JY, Zhang JH, Zhang HS, Teng S (2012). Genetic analysis of rice germination tolerance to flooding. Chinese Bulletin of Botany.

[CR6] Duan YDY, Li FLF, Li YLY, Tang YTY, Kong XKX, Feng ZFZ, Anthony TATG, Watford MWM, Hou YHY, Wu GWG (2016). The role of leucine and its metabolites in protein and energy metabolism. Amino Acids.

[CR7] Galili G, Avin-Wittenberg T, Angelovici R, Fernie AR (2014). The role of photosynthesis and amino acid metabolism in the energy status during seed development. Front Plant Sci.

[CR8] Ghosal S, Casal C, Quilloy FA, Septiningsih EM, Mendioro MS, Dixit S (2019) Deciphering genetics underlying stable anaerobic germination in rice: phenotyping, QTL identification, and interaction analysis. Rice 12(1) 10.1186/s12284-019-0305-y PMC6629739 31309351

[CR9] Gipson AB, Morton KJ, Rhee RJ, Simo S, Clayton JA, Perrett ME, Binkley CG, Oakes DL, Rouhier MF, Rouhier KA (2017). Disruptions in valine degradation affect seed development and germination in Arabidopsis. Plant J.

[CR10] Gommers CMM, Monte E (2017) Seedling establishment: a dimmer switch-regulated process between dark and light signaling. Plant Physiol. 1460–2017 10.1104/pp.17.01460 PMC5813566 29217596

[CR11] Harper AE, Miller RH, Block KP (1984) Branched-chain amino acid metabolism. Annual Rev Nutrit. 409–454 10.1146/annurev.nu.04.070184.002205 6380539

[CR12] Hofmann NR (2016) Opposing functions for plant xanthine dehydrogenase in response to powdery mildew infection: production and scavenging of reactive oxygen species. Plant Cell (5): 1001 10.1105/tpc.16.00381 PMC4904684 27166140

[CR13] Howell KA, Cheng K, Murcha MW, Jenkin LE, Millar AH, Whelan J (2007). Oxygen initiation of respiration and mitochondrial biogenesis in rice. J Biol Chem.

[CR14] Hsu S, Tung C (2015) Genetic mapping of anaerobic germination-associated QTLs controlling coleoptile elongation in rice. Rice 8(1) 10.1186/s12284-015-0072-3 PMC4689725 26699727

[CR15] Hsu SHSS, Tung CTCS (2017) RNA-Seq analysis of diverse rice genotypes to identify the genes controlling coleoptile growth during submerged germination. Front Plant Sci 762 10.3389/fpls.2017.00762 PMC5430036 28555145

[CR54] Huang XH, Wei XH, Sang T (2010) Genome-wide association studies of 14 agronomic traits in rice landraces. Nat Genet 42(11):961–967 10.1038/ng.695 20972439

[CR16] Jeong JM, Cho YC, Jeong JU, Mo YJ, Kim CS, Kim WJ, Baek MK, Kim SM (2020). QTL mapping and effect confirmation for anaerobic germination tolerance derived from thejaponica weedy rice landrace PBR. Plant Breed.

[CR17] Kato-Noguchi H, Ohashi C (2006) Effects of anoxia on amino acid levels in rice coleoptiles. Plant Product Sci (4): 383–387

[CR18] Kato-Noguchi H, Yasuda Y, Sasaki R (2011) Anoxia tolerance and sugar level in roots and coleoptiles of aerobically germinated rice. Plant Product Sci (4): 325–330

[CR19] Kim J, Mo Y, Ha SK, Kim WJ, Kim BK, Jeung JU, Jeong JM (2019). QTL analysis for tolerance to anaerobic germination in japonica rice (*Oryza Sativa* L.). Korean J Breed Sci.

[CR20] Langfelder P, Horvath S (2008) WGCNA: an R package for weighted correlation network analysis. BMC Bioinf (1): 1–13 10.1186/1471-2105-9-559 PMC2631488 19114008

[CR21] Lasanthi-Kudahettige R, Magneschi L, Loreti E, Gonzali S, Perata P (2007). Transcript profiling of the anoxic rice coleoptile. Plant Physiol.

[CR22] Lee TM, Chu C (1992). Ethylene-Induced Polyamine Accumulation in Rice (*Oryza sativa* L.) Coleoptiles. Plant Physiol.

[CR23] Lee KW, Chen PW, Lu CA, Chen S, Ho T, Yu SM (2009). Coordinated responses to oxygen and sugar deficiency allow rice seedlings to tolerate flooding. Science Signaling.

[CR24] Li DX. (2019) QTL mapping of seedling tolerance traits in rice seeds. Master's thesis of South China Agricultural University

[CR52] Li XX, Chen Z, Zhang GM (2020) Analysis of genetic architecture and favorable allele usage of agronomic traits in a large collection of Chinese rice accessions. Sci China Life Sci 63(11):1688–1702 10.1007/s11427-019-1682-6 32303966

[CR25] Liu L, Li X, Liu S, Min J, Liu W, Pan X, Fang B, Hu M, Liu Z, Li Y, Zhang H (2021). Identification of QTLs associated with the anaerobic germination potential using a set of Oryza nivara introgression lines. Genes Genom.

[CR26] Liu LC, Li XX, Li XC, Pan XW, Min J, Liu WQ, Hu M, Duan YH, Yu YY, Zhang HQ (2021). Genome-wide association analysis of anaerobic germination tolerance of rice seeds. J Plant Genet Resour.

[CR27] Love MI, Huber W, Anders S (2014). Moderated estimation of fold change and dispersion for RNA-seq data with DESeq2. Genome Biol.

[CR53] Lu Q, Zhang M, Niu X (2015) Genetic variation and association mapping for 12 agronomic traits in indica rice. BMC Genom 16(1):1067 10.1186/s12864-015-2245-2 PMC4681178 26673149

[CR55] Mather KA, Caicedo AL, Polato NR (2007) The extent of linkage disequilibrium in rice (*Oryza sativa* L.). Genetics 177(4):2223–2232 10.1534/genetics.107.079616 PMC2219496 17947413

[CR28] Miyashita Y, Dolferus R, Ismond KP, Good AG (2007). Alanine aminotransferase catalyses the breakdown of alanine after hypoxia in Arabidopsis thaliana. Plant J Cell Mol Biol.

[CR29] Mustroph A, Boamfa EI, Laarhoven LJJ, Harren FJM, Albrecht G, Grimm B (2006). Organ-specific analysis of the anaerobic primary metabolism in rice and wheat seedlings. I: Dark ethanol production is dominated by the shoots. Planta.

[CR30] Narsai R, Howell KA, Carroll A (2009). Defining core metabolic and transcriptomic responses to oxygen availability in rice embryos and young seedlings. Plant Physiol.

[CR31] Reggiani R, Cantu CA, Brambilla I, Bertani A (1988). Accumulation and interconversion of amino acids in rice roots under anoxia. Plant Cell Physiol.

[CR32] Reggiani R, Hochkoeppler A, Bertani A (1989). Polyamines in rice seedlings under oxygen-deficit stress. Plant Physiol.

[CR33] Ricoult C, Echeverria LO, Cliquet J, Limami AM (2006). Characterization of alanine aminotransferase (AlaAT) multigene family and hypoxic response in young seedlings of the model legume Medicago truncatula. J Exper Botany.

[CR34] Rohilla M, Singh N, Mazumder A, Sen P, Roy P, Chowdhury D (2020). Genome-wide association studies using 50 K rice genic SNP chip unveil genetic architecture for anaerobic germination of deep-water rice population of Assam. India Mol Genet Genomics.

[CR35] Sachs MM, Freeling M, Okimoto R (1980). The anaerobic proteins of maize. Cell.

[CR36] Sachs MMSA, Subbaiah CCSB, Saab INSB (1996). Anaerobic gene expression and flooding tolerance in maize. J Exper Botany.

[CR37] Scofield GN, Aoki N, Hirose T, Takano M, Jenkins CL, Furbank RT (2007). The role of the sucrose transporter, OsSUT1, in germination and early seedling growth and development of rice plants. J Exper Botany.

[CR38] Septiningsih EM, Ignacio JCI, Sendon PMD, Sanchez DL, Ismail AM, Mackill DJ (2013). QTL mapping and confirmation for tolerance of anaerobic conditions during germination derived from the rice landrace Ma-Zhan Red. Theor Appl Genet.

[CR39] Su L, Yang J, Li D, Peng Z, Guo T (2021) Dynamic genome-wide association analysis and identification of candidate genes involved in anaerobic germination tolerance in rice. Rice 14(1) 10.1186/s12284-020-00444-x PMC7788155 33409869

[CR40] Sup ABS, Sup FMS, Sup RBS (1981). Some effects of anaerobiosis on protein metabolism in rice roots. Zeitschrift für Pflanzenphysiologie.

[CR41] Tadege M, Dupuis I, Kuhlemeier C, Tadege M, Dupuis I, Kuhlemeier C (1999). Ethanolic fermentation: new functions for an old pathway. Trends Plant Sci.

[CR42] Taylor NL, Howell KA, Heazlewood JL, Tan TYW, Narsai R, Huang S, Whelan J, Millar AH (2010). Analysis of the rice mitochondrial carrier family reveals anaerobic accumulation of a basic amino acid carrier involved in arginine metabolism during seed germination. Plant Physiol.

[CR43] Usadel B (2005). Extension of the visualization tool MapMan to allow statistical analysis of arrays, display of corresponding genes, and comparison with known responses. Plant Physiol.

[CR44] Vartapetian BB, Jackson MB (1997). Plant adaptations to anaerobic stress. Annals Botany.

[CR45] Voesenek LACJ, Bailey-Serres JSS (2009). Plant biology: genetics of high-rise rice. Nature.

[CR46] Yan N, Du Y, Liu X, Chu M, Shi J, Zhang H, Liu Y, Zhang Z (2019). A comparative UHPLC-QqQ-MS-based metabolomics approach for evaluating Chinese and North American wild rice. Food Chem.

[CR47] Yang P, Li X, Wang X, Chen H, Shen S (2010). Proteomic analysis of rice (*Oryza sativa*) seeds during germination. Proteomics.

[CR48] Yang J, Sun K, Li D, Luo L, Liu Y, Huang M, Yang G, Liu H, Wang H, Chen Z, Guo T (2019). Identification of stable QTLs and candidate genes involved in anaerobic germination tolerance in rice via high-density genetic mapping and RNA-Seq. BMC Genom.

[CR49] Yu S, Lee H, Lo S, Ho TD (2020). How does rice cope with too little oxygen during its early life?. New Phytologist.

[CR50] Zhang M, Lu Q, Wu W, Niu X, Wang C, Feng Y, Xu Q, Wang S, Yuan X, Yu H, Wang Y, Wei X (2017). Association mapping reveals novel genetic loci contributing to flooding tolerance during germination in indica rice. Front Plant Sci.

[CR51] Zhang HL, Chen YY, Yang LX, Shen YB (2020). Regulation of γ-aminobutyric acid on plant growth and development and stress resistance. Plant Physiol J.

