# Supplementary material for: GWAS Combined with WGCNA of Transcriptome and Metabolome to Excavate Key Candidate Genes for Rice Anaerobic Germination
Source: Rice (N Y). 2023 Oct 31;16:49. doi: 10.1186/s12284-023-00667-8 (PMC10618154; doi:10.1186/s12284-023-00667-8)
Supplement: Supplementary file 14 — Additional file 14. Supporting Information. Supplementary materials and methods. [file 12284_2023_667_MOESM14_ESM.docx]

**Supporting Information**

**Supplementary Materials and Methods**

**Plant materials and treatment conditions**

The seeds were placed in an oven at 50°C for 7 d to break dormancy. Plump and healthy seeds were picked for manual peeling. Aerobic treatment was performed by placing the seeds in a 9 cm Petri dish lined with two layers of circular qualitative filter paper and adding 10 ml of sterile distilled water. The Petri dishes were placed in an incubator with a temperature of 30°C and a light cycle of 8 h light/16 h dark for 2, 3, and 4 days of germination. Hypoxic treatment was performed by placing the seeds into a transparent flat-bottom centrifuge tube with a volume of 50 ml and a height of 10 cm, and the centrifuge tube was filled with distilled water to create a hypoxic environment. All the centrifuge tubes were covered and placed in an incubator with a light cycle of 8 h light/16 h dark for 2, 3, and 4 days. In addition, two treatments for condition conversion were set up during the germination process: after 3 d of aerobic germination, the aerobic germination material was transferred to hypoxic conditions for 1 d (A3dAN1d); similarly, after 3 d of hypoxic germination, the hypoxic germination material was transferred to aerobic conditions for 1 d (AN3dA1d).

For broadly targeted metabolomic analysis, material was sampled (all visible tissues) at 0 d, 2 d, 3 d, 4 d and two transition time points (A3dAN1d and AN3dA1d) under aerobic and hypoxic conditions. For dynamic widely targeted metabolome research, two materials were used, with 3 replicates per treatment, for a total of 54 samples. For dynamic targeted metabolomics and transcriptomics, material was sampled (all visible tissues) at 4 d of aerobic and hypoxic conditions and at two transition time points. Two types of materials were subjected to mixed sampling, with 3 replicates per treatment, for a total of 24 samples. The samples were quickly frozen in liquid nitrogen and stored at -80°C.

The WinRHIZO (Regent Instruments Inc., Québec, Canada) root image analysis system was used to measure the phenotypic data of the coleoptiles (CL, CSA, CV and CD).

**Dynamic widely targeted metabolite detection and data analysis**

After the samples were vacuum freeze-dried, the samples were ground into powder, and 100 mg of the powder was weighed with an analytical balance, dissolved in 1.0 mL of extract, and vortexed every 10 min, which was repeated three times; then, the samples were placed in a 4°C refrigerator. After overnight centrifugation (10,000 ×g, 10 min), the supernatant was aspirated, and the samples were filtered through a microporous membrane (0.22 μm pore size) and stored in a sample vial for LC‒MS/MS analysis.

The data acquisition instrument system mainly included ultrahigh-performance liquid chromatography (UPLC) (Shim-pack UFLC SHIMADZU CBM30A, http://www.shimadzu.com.cn/) and tandem mass spectrometry (MS/MS) (Applied Biosystems 6500 QTRAP, http://www.appliedbiosystems.com.cn/).

Metabolite differences between samples were analyzed by the orthogonal partial least squares-discriminant analysis (OPLS-DA) method using R language (Thévenot et al., 2015). If a metabolite had VIP (variable importance in projection) ≥ 1 and P < 0.05 (t test), the metabolite was considered to be a differentially abundant metabolite between the two groups.

**Transcriptomic Sequencing**

Validation of candidate genes by real-time quantitative RT‒PCR. The reverse transcription kit used in this study was an EVO M-MLV RT kit (Aikere, #AG11728), and a SYBR® green premix pro taq HS qPCR kit (Aikere, #AG11728) was used for qRT‒PCR. The rice housekeeping gene (OseEF1α) was used as an internal reference to determine the mRNA expression level of candidate genes. Three biological technical replicates were set for each sample. The relative expression levels of genes were calculated using the 2^-ΔΔct^ method. Gene-specific primers were designed by NCBI primer BLAST (http://www.ncbi.nlm.nih.gov/tools/primer-blast/). The primer sequences of the candidate genes are listed in Table S2.

**Weighted gene coexpression network analysis and core metabolite selection**

The soft threshold is one of the most critical parameters and mainly affects the independence and average connectivity of coexpression modules. To determine the optimal value of the soft threshold so that the adjacency function could better satisfy the scale-free condition, the logarithm of the number of connected nodes (log(i)) and the logarithm of the occurrence probability of this node (log(p(i))) were negatively correlated, and the minimum value when the correlation coefficient reached a plateau (or greater than 0.8) was used as the soft threshold β for subsequent analysis.

**Genome-Wide Association analysis (GWAS)**

The population used for genome-wide association analysis (GWAS) consisted of 591 rice germplasm, and the genomes of all 591 accessions were sequenced on the Illumina HiSeq 2500 Sequencing Systems Platform (Illumina Inc. USA). Seeds of the population were planted in a paddy field at South China Agricultural University, Guangzhou, China (at approximately 113° east longitude and approximately 23° north latitude) at the late cropping season (LS) in 2019. Each variety was planted in a block designed of 6 column × 6 row, with spacing of 20 cm among the plants. Crop management, and disease and insect pest control were performed as locally recommended. All the materials come from the germplasm resource bank of the National Engineering Research Center of Plant Space Breeding. Considering that seed maturity affects AG, six individual plants in the middle of each block were harvested independently at the 40th day after heading in LS. The harvested seeds were dried in a heated air dryer at 42 °C for 5 d and then stored at -20 °C. After the seeds were placed in an oven at 50°C for 7 days to break dormancy, coleoptile length (CL), coleoptile surface area (CSA), coleoptile volume (CV), and coleoptile diameter (CD) of the materials were measured after 2d, 3d and 4d treatment under hypoxic conditions, and genome-wide association analysis was conducted using the data of the coleoptile of these four traits.

**Construction of *OsAlaAT1* transgenic vector and acquisition of transgenic materials**

**Construction of CRISPR/Cas9 knockout vector**

According to *OsAlaAT1* CDS of DNA sequences, we use http://skl.scau.edu.cn/home/ for CRISPR/Cas9 knock out targeted by design and selection, and with the aim to template amplification pGTR plasmid middle segment, Then, T7 ligase and Bsa I endonucliase were used for side cutting and side linking, and the ligand products were further amplified by PCR to obtain tRNA-Target site-gRNA fragments, which were then treated by Fok I endonucliase and linked to the expression vector pRGEB32, which was ligated by Bsa I enzyme, by T4 ligase. And transformed Escherichia coli DH5α. The successful construction of the vector was confirmed by colony PCR, target fragment sequencing and enzyme digestion.

**Construction of overexpression vector**

Primers were designed according to DNA sequences of and *OsAlaAT1* coding region. First, full-length CDS containing terminator of *OsAlaAT1* were obtained by using ZH11 cDNA as template. The target fragment was then homologous recombined with the linearized vector cut by Hind III and BamHⅠ, and then connected to the receptive state of Escherichia coli DH5α. Monoclonal shaken bacteria were selected, and the positive clones were preliminarily determined by colony PCR after the bacterial liquid was stored. The positive clones were further sequenced by plasmid extraction and sequencing, and the successful construction of the overexpression vector was confirmed after the sequence alignment showed no variation.

**Construction of promoter fusion GUS expression vector**

Using ZH11 DNA as template, the upstream 2 kb sequence of ATG was amplified as promoter. SacI and NcoI were used for enzyme digestion of the target fragment and vector, respectively. After gum recovery, the target fragment was inserted into pCAMBIA1305.1 vector through homologous recombination. After transformation of Escherichia coli, positive clones were selected for shaken bacteria, colony PCR, enzyme digestion and sequencing were used to confirm the successful construction of vector.

The knockout, overexpression and GUS fusion expression vector were transformed by the japonica Zhonghua 11 (ZH11). The transgenic plants were obtained by the rapid transformation method mediated by Agrobacterium tumefaciens. The transformation was completed by Wuhan biorun biosciences co.,ltd.

**Statistical analysis**

The phenotypic data of coleoptiles are presented as the mean ± standard error of the mean. The correlation between the phenotype of the coleoptile and the selected core metabolites was analyzed by Pearson correlation (SPSS, version 23.0). P < 0.05 was considered a statistically significant difference. Multivariate statistical analyses and partial least squares discriminant analysis (PLS) were carried out using SIMCA-P software (version 11.5, Umetrics, Sweden).

The Kyoto Encyclopedia of Genes and Genomes (KEGG) Pathway database (http://www.kegg.jp/kegg/pathway.html) is centered on metabolic reactions and concatenates possible metabolic pathways and corresponding regulatory proteins (Xia et al., 2015). For metabolites in each module, KEGG pathway enrichment analysis was conducted to analyze the biological functions of the modules. Significantly enriched pathways in which the metabolites in a module were involved were compared to the background and defined by a hypergeometric test and a threshold of P value less than 0.05.

**Qualitative and quantitative analyses of metabolites and quality control (QC) analysis of samples**

A typical TIC plot of one QC sample is shown in Fig. S1A; the TIC plot represents a continuous description of the intensity sum of all ions in the mass spectrum at different time points. A multipeak detection plot of metabolites in MRM mode is illustrated in Fig. S1B; the figure shows the ion current plot of multiple substances, where the abscissa indicates the RT of the metabolites and the ordinate indicates the ion current intensity in counts per second (cps). Based on the local metabolite database, qualitative and quantitative mass spectrometry analyses were conducted on the metabolites in the samples, as shown in Fig. 2B. The multipeak detection plot of metabolites in MRM mode revealed the substances that were detected in the samples. In the plot, each peak in a different color represents a detected metabolite; information on the metabolite serial number, name, class, molecular weight (Da) and KEGG ID is listed in Table S3. In total, 730 metabolites, comprising alcohols and polyols, alkaloids, amino acid derivatives, amino acids, anthocyanins, benzoic acid derivatives, and carbohydrates, were identified (Table S4).

The quality control sample (QC) was prepared by mixing sample extracts and was used to analyze the repeatability of the sample under the same treatment method. During analysis with the instrument, a quality control sample is inserted between every 10 test analysis samples to monitor the repeatability of the analysis process. An overlay analysis was applied to the TIC plots of different QC samples to evaluate the repeatability of metabolite extraction and detection, namely, the technical repeatability. Fig. S2 shows an overlay of the TIC plots between the first and last QC samples, indicating that the TIC plots of the metabolites had a high degree of overlap. This result suggests that the RT and peak intensities were consistent between the two QC samples, signifying good signal stability in the detection of the same sample at different times. Therefore, these results indicated that the data recorded in this study have good repeatability and reliability.

**Principal component analysis (PCA) for the** [**Aerobic**](file:///C:\Program%2520Files%2520(x86)\Youdao\Dict\8.3.1.0\resultui\html\index.html#/javascript:;) **group versus the** [**Anaerobic**](file:///C:\Program%2520Files%2520(x86)\Youdao\Dict\8.3.1.0\resultui\html\index.html#/javascript:;) **group in the two types of directly seeded rice**

Principal component analysis (PCA) was performed on samples (including QC samples) to gain a preliminary understanding of the overall metabolic differences between samples in each group and the degree of variability among samples within a group (Chen et al., 2009). The PCA scatter plots of all the samples (including QC samples) are shown in Fig. S3. PCA clearly separated the two species and oxygen treatments, where the first principal component (pc1) separated the different oxygen treatment groups, and its contribution value reached 36.68%; the second principal component (pc2) separated the two varieties, and its contribution value reached 14.28%. This led us to further confirm that the different oxygen environments were the main factor affecting the germination of these two rice varieties.
