# Supplementary material for: GWAS Combined with WGCNA of Transcriptome and Metabolome to Excavate Key Candidate Genes for Rice Anaerobic Germination
Source: Rice (N Y). 2023 Oct 31;16:49. doi: 10.1186/s12284-023-00667-8 (PMC10618154; doi:10.1186/s12284-023-00667-8)
Supplement: Supplementary file 1 — Additional file 1. Fig. S1. Frequency distribution and Manhattan map and quantile-quantile (QQ) plots of SNPs associated with coleoptile diameter(CD) at different time points under anaerobic germination. Fig. S2. Frequency distribution and Manhattan map and quantile-quantile (QQ) plots of SNPs associated with coleoptile length(CL) at different time points under anaerobic germination. Fig. S3. Frequency distribution and Manhattan map and quantile-quantile (QQ) plots of SNPs associated with coleoptile surface area(CSA) at different time points under anaerobic germination. Fig. S4. Frequency distribution and Manhattan map and quantile-quantile (QQ) plots of SNPs associated with coleoptile volum(CV) at different time points under anaerobic germination. Fig. S5. Evolutionary tree analyses of 591 rice natural populations structure. Fig. S6. Estimation of genome-wide average LD decay distance. Fig. S7. Total ion current of one quality control sample by mass spectrometry detection (a) and multi-peak detection plot of metabolites in the multiple reaction monitoring mode (b). Fig. S8. Overlapping total ion flow chart (TIC chart) for QC sample mass spectrometry detection. Fig. S9. Principal component analysis (PCA) result. Fig. S10. Categorization of metabolites detected. Fig. S11. Sample Hierarchical Clustering Tree. Fig. S12. Power value curve. Analysis of network topology for various soft thresholding power. Fig. S13. Hierarchical cluster tree. Fig. S14. The expression patterns of metabolites in each module. Fig. S15. Correlation heatmap between samples. Fig. S16. Principal component analysis (PCA) result. Fig. S17. Eighteen modules heatmap of gene expression patterns. Fig. S18. Module partitioning and metabolite correlation heatmaps. Fig. S19. GO and KEGG enrichment analysis of dark green, dark gray, and light green modules. Fig. S20. qRT‒PCR results of OsAlaAT1, OsGAD4, Os09g0424600 and Os06g0665500 (OsAAH). Fig. S21. The spatio-temporal expression of LOC_Os10g25130 in Ni [file 12284_2023_667_MOESM1_ESM.docx]

**Supplementary Fig. S1-S21**

**Fig. S1** Frequency distribution and Manhattan map and quantile-quantile (QQ) plots of SNPs associated with coleoptile diameter(CD) at different time points under anaerobic germination. The left, middle and right of the picture are: frequency square distribution diagram, Manhattan diagram, QQ diagram. The horizontal dashed line represents the significance threshold -log10(P) = 5.00. And Fig. S2-S4 is the same.

**Fig. S2** Frequency distribution and Manhattan map and quantile-quantile (QQ) plots of SNPs associated with coleoptile length(CL) at different time points under anaerobic germination.

**Fig. S3** Frequency distribution and Manhattan map and quantile-quantile (QQ) plots of SNPs associated with coleoptile surface area(CSA) at different time points under anaerobic germination.

**Fig. S4** Frequency distribution and Manhattan map and quantile-quantile (QQ) plots of SNPs associated with coleoptile volum(CV) at different time points under anaerobic germination.

**Fig. S5** Evolutionary tree analyses of 591 rice natural populations structure. GJ means *japonica*, XI means *indica* ,and POP1-POP4 are three *indica* rice subgroups.

**Fig. S6** Estimation of genome-wide average LD decay distance

**Fig. S7** Total ion current of one quality control sample by mass spectrometry detection (a) and multi-peak detection plot of metabolites in the multiple reaction monitoring mode (b).

**Fig. S8** Overlapping total ion flow chart (TIC chart) for QC sample mass spectrometry detection. The detection in the positive ion mode (a). The detection in negative ion mode (b).

**Fig. S9** Principal component analysis (PCA) result. The abscissa and the ordinate represent the scores of pc1 and pc2, respectively; the blue ring represents 02428 (*Japonica.*); the red ring is YZX (*Indica.*), The mix is the quality control sample mentioned above.

**Fig. S10** Categorization of metabolites detected. i, total metabolites. ii and iii represent up-regulated and down-regulated metabolites during aerobic germination, respectively. iv and v represent up-regulated and down-regulated metabolites during anaerobic germination, respectively. vi and vii represent up-regulated and down-regulated metabolites during anaerobic germination when compared to aerobic germination (A v AN), respectively.

**Fig. S11** Sample Hierarchical Clustering Tree. All samples were clustered according to the expression levels of all genes to analyze sample relationships.

**Fig. S12** Power value curve. Analysis of network topology for various soft thresholding power. The panel(a) shows the scale-free fit index (y-axis) as a function of the soft thresholding power (x-axis). The panel (b) displays the mean connectivity (degree, y-axis) as a function of the soft thresholding power (x-axis).

**Fig. S13** Hierarchical cluster tree. Clustering dendrograms of metabolites, with dissimilarity based on topological overlap, together with assigned module colors. As a result, 11 co-expression modules were constructed and was shown in different color. These modules were ranged from large to small by the number of metabolites they included.

**Fig. S14** The expression patterns of metabolites in each module. The expression heatmap of each module shows the expression of each metabolite in the module in different samples; red indicates upregulation, and green indicates downregulation; below the expression heatmap is the module eigenvalues of each module in different sample models.

**Fig. S15** Correlation heatmap between samples.

**Fig. S16** Principal component analysis (PCA) result. HA3dAN1d, HA4d, HAN3dA1d and HAN4d represent that aerobic treatment for 3 days was transferred to hypoxia treatment for 1 day, aerobic treatment for 4 days, hypoxia treatment for 3 days was transferred to aerobic treatment for 1 day and hypoxia treatment for 1 day in high pools, respectively. LA3dAN1d, LA4d, LAN3dA1d and LAN4d represent that aerobic treatment for 3 days was transferred to hypoxia treatment for 1 day, aerobic treatment for 4 days, hypoxia treatment for 3 days was transferred to aerobic treatment for 1 day and hypoxia treatment for 1 day in low pools, respectively.

**Fig. S17** Eighteen modules heatmap of gene expression patterns. The expression pattern of each gene contained in each module is displayed with a heat map, and the change of the eigenvalues of the module in different samples is presented by a histogram (equivalent to the module expression pattern). In the figure, the upper figure is the heat map of the expression of genes in the module in different samples, red is up-regulated, green is down-regulated; the lower figure is the module eigenvalues in different samples. The grey module has fewer genes and cannot form a heatmap.

**Fig. S18** Module partitioning and metabolite correlation heatmaps. a, Modules divided according to clustering results. b, Heatmap of metabolite and module correlations.

**Fig. S19** GO and KEGG enrichment analysis of dark green, dark gray, and light green modules. a is the dark green module, b is the dark gray module, and c is the GO and KEGG enrichment analysis of the light green module. i) GO enrichment analysis, ii) top 20 enriched KEGG pathways.

**Fig. S20** qRT‒PCR results of *OsAlaAT1*, *OsGAD4*, *Os09g0424600* and *Os06g0665500* (*OsAAH*).

**Fig. S21** The spatio-temporal expression of LOC_Os10g25130 in Nipponbare based on RAP-DB and BAR database. The organs and tissues including leaf blade, leaf sheath, root, stem, inflorescence, anther, pistil, lemma, palea, ovary, embryo, endosperm. DAF, days after fertilization. RAP-DB, rice annotation project database; BAR, bio-analytic resource for plant biology.
